# The Novel *Candida albicans* Transporter Dur31 Is a Multi-Stage Pathogenicity Factor

**DOI:** 10.1371/journal.ppat.1002592

**Published:** 2012-03-15

**Authors:** François L. Mayer, Duncan Wilson, Ilse D. Jacobsen, Pedro Miramón, Katharina Große, Bernhard Hube

**Affiliations:** 1 Department of Microbial Pathogenicity Mechanisms, Hans-Knoell-Institute, Jena, Germany; 2 Friedrich Schiller University, Jena, Germany; UCSF, United States of America

## Abstract

*Candida albicans* is the most frequent cause of oral fungal infections. However, the exact pathogenicity mechanisms that this fungus employs are largely unknown and many of the genes expressed during oral infection are uncharacterized. In this study we sought to functionally characterize 12 previously unknown function genes associated with oral candidiasis. We generated homozygous knockout mutants for all 12 genes and analyzed their interaction with human oral epithelium *in vitro*. Eleven mutants caused significantly less epithelial damage and, of these, deletion of orf19.6656 (*DUR31*) elicited the strongest reduction in pathogenicity. Interestingly, *DUR31* was not only involved in oral epithelial damage, but in multiple stages of candidiasis, including surviving attack by human neutrophils, endothelial damage and virulence *in vivo*. *In silico* analysis indicated that *DUR31* encodes a sodium/substrate symporter with 13 transmembrane domains and no human homologue. We provide evidence that Dur31 transports histatin 5. This is one of the very first examples of microbial driven import of this highly cytotoxic antimicrobial peptide. Also, in contrast to wild type *C. albicans*, *dur31*Δ/Δ was unable to actively increase local environmental pH, suggesting that Dur31 lies in the extracellular alkalinization hyphal auto-induction pathway; and, indeed, *DUR31* was required for morphogenesis. In agreement with this observation, *dur31*Δ/Δ was unable to assimilate the polyamine spermidine.

## Author Summary

The normally commensal yeast, *Candida albicans* is the leading cause of both oral and vaginal thrush, as well as life-threatening disseminated infections in the nosocomial setting. Treatment of such infections is hampered by the limited number of suitable antimycotics. Therefore, understanding how this fungus causes disease is crucial for the development of more effective diagnostic tools and therapeutic strategies. Here we identified a novel *C. albicans* transporter that has no human homologue and demonstrate its role during multiple stages of candidiasis. We also show that this transporter is involved in modifying environmental pH and in filamentous growth. Additionally, deletion of *DUR31* rendered cells unable to assimilate a polyamine (spermidine) as a nutrient source, but resistant to the antimicrobial peptide histatin 5. Therefore, Dur31 has multiple transport functions, which can be either beneficial or detrimental to the fungus.

## Introduction

The human oral cavity represents a discrete environmental niche, harboring a diverse and complex microbiome. In up to 80% of healthy individuals the fungus *Candida albicans*, and to a lesser extent other *Candida* spp. are part of this oral microbiome where they usually exist as harmless commensals [1]–[3]. However, disturbances in the human immune status and other predisposing factors can allow the fungus to switch from a commensal to a pathogen, causing oral infections (oral candidiasis). For example, denture wearing, reduced salivary flow and extremes of age are risk factors [2]. HIV^+^/AIDS patients in particular are predisposed to oral candidiasis with as many as 80–90% suffering from recurrent infections [4]–[7]. In order to persist as part of the human oral microbiome and as a prerequisite for infection, *C. albicans* must adhere to other microbes and/or to epithelial host cells. Adherence is mediated mainly by adhesins, including the hyphal wall protein 1 (Hwp1) and members of the agglutinin-like sequence (Als) family [8]. The corresponding genes were found to be upregulated during oral candidiasis [9]. Besides adhesins, hydrophobicity and the interaction between pathogen-associated molecular patterns (PAMPs) and pattern recognition receptors (PRRs) on host cells also mediate adhesion [10]. The initiation of oral infections is associated with the formation of elongated fungal filaments (hyphae) which can penetrate into the oral epithelium. This invasion process can occur via two distinct mechanisms, induced endocytosis and active penetration [9], [11]–[13]. Induced endocytosis does not depend on fungal viability and is characterized by engulfment of the fungal cell by the host cell [8], [9], [11]. This process is initiated by binding of the host cell cadherins, N-cadherin (endothelial cells) and E-cadherin (epithelial cells), to the fungal invasins Als3 [14] and Ssa1 [15]. Active penetration is dependent upon fungal viability and involves direct penetration of *C. albicans* hyphae into host cells or at intercellular junctions [8], [11]. This process is believed to be driven by mechanical pressure of the invading hyphal tip and the secretion of hydrolytic enzymes. Following these adhesion and invasion events, the fungus damages epithelial host cells, mediated by a combination of active penetration, hyphal extension and the expression of largely unknown virulence factors for deeper tissue invasion and further inter-epithelial invasion [12]. Importantly, we recently demonstrated that adhesion and invasion events alone do not result in host cell damage [12], suggesting that other, yet unidentified, activities play a role in tissue destruction. The morphogenetic switch is believed to be a key virulence factor because mutants which are impaired in filament-formation are avirulent [16]. Filamentation is induced by multiple environmental cues such as temperature, pH, CO_2_, or contact to epithelial and endothelial host cells [9], [12]. Recently, it has been shown that *C. albicans* is able to auto-induce filament formation by actively alkalinizing its extracellular environment [17]. In an acidic environment the fungus can raise the extracellular pH from 4 to >7 within 12 h, thereby triggering the yeast-to-hypha transition [17]. Alkalinization occurs in glucose-limited media and depends on the presence of exogenous amino acids [17]. By screening around 500 mutant strains and performing transcriptional profiling, the authors demonstrated that the amino acid permease regulator Stp2, the acetyl-coenzyme A hydrolase Ach1, the urea amidolyase Dur1,2 and the putative ammonia exporter Ato5 are required for extracellular alkalinization [17]. The authors concluded that under nutrient limitation, *C. albicans* assimilates amino acids as carbon source, exports the amine group in the form of ammonia and thereby raises extracellular pH, which in turn results in the yeast-to-hyphal transition [17].

One of the key strategies that oral epithelial cells employ to defend themselves against *C. albicans* infections is the production of antimicrobial peptides like cathelicidins [18], [19], defensins [20], and histatins [21], [22]. Among the family of histatins, histatin 5 has the highest killing efficacy against *C. albicans*. Additionally, in deeper tissues and in blood, neutrophils exhibit key defense activities as part of the innate immune system and have been shown to be central in killing *C. albicans* [23]. Importantly, in certain high risk patients [24], *C. albicans* can cause life-threatening systemic infections [25]. Approximately 30–37% of patients suffering from systemic candidiasis die during the course of the infection [26], [27]. Interestingly, HIV infection is not an independent risk factor for disseminated candidiasis, suggesting that the two diseases might develop independently from each other [8]. In a previous study we performed genome-wide transcriptional profiling of samples from HIV^+^ patients with oral candidiasis and of *in vitro* oral infection models, and identified genes encoding known and unknown-function proteins associated with oral infection [9]. These investigations led to the discovery of the novel infection-associated gene *EED1*, which appears to encode a key regulator of hyphal extension [9], [28].

The aim of the current study was to identify further novel oral infection-associated genes in *C. albicans*. We therefore selected a set of 12 previously uncharacterized genes, based on their transcriptional upregulation during oral infection [9] and *in silico* functional predictions. We generated knockout mutants for all 12 genes and show that 11 mutants were significantly attenuated in their capacity to damage oral host cells *in vitro*. We then focused our investigations on the characterization of the novel gene *DUR31* which encodes a predicted plasma membrane localized sodium substrate transporter with no homologue in humans. We demonstrate a crucial role for Dur31 in host cell damage, resistance to neutrophils and virulence. Finally we provide mechanistic insight into the role of Dur31 in polyamine assimilation, histatin 5 import and extracellular alkalinization induced hyphal formation.

## Results

### Identification of *C. albicans* genes associated with human oral infections

The first objective of this study was to identify novel and previously uncharacterized *C. albicans* genes, associated with oral candidiasis. We hypothesized that *C. albicans* genes specifically upregulated during oral infection represent promising candidates for novel, oral infection-associated virulence factors. Based on previously published transcriptional data of *C. albicans* during both infection of the oral cavity of HIV^+^ individuals and infection of reconstituted human oral epithelium [9], we identified 12 genes that were upregulated at least two-fold under one or both conditions (Table S1). We performed *in silico* analysis of all 12 genes and found nine to contain one or more transmembrane domain(s) in their predicted protein sequences (Table 1). Using a targeted gene deletion strategy we then generated homozygous knockout mutants for each of the twelve unknown function genes (Table 2).

**Table 1:** In silico analysis of *C. albicans* oral infection upregulated unknown function genes.

Gene name	Common name	Motif	Localization	Human homologue
orf19.1150	-	Zinc finger domain	nucl	Transcription factor GATA-4 (14%)
orf19.1353	-	3 TMs	plas	-
orf19.2959.1	-	2 TMs	mito	-
orf19.3617	*GTR1*	1 coiled coil region	mito	Ras-related GTP-binding protein A (52%)
orf19.3872	-	5 TMs	plas	-
orf19.5443	*BNA4*	1 TM	cyto	Kynurenine 3-monooxygenase (35%)
orf19.5848	-	2 TMs, zinc finger RING domain	ER	E3 ubiquitin-protein ligase precursor (13%)
orf19.6200	-	1 TM	extr	Peptidase inhibitor 16 precursor (8%)
orf19.6656	*DUR31* 1 (*DUR3*)^2^	13 TMs, Na^+^-substr- symp domain	plas	-
orf19.6847	-	-	nucl	Methyltransferase like-protein 19 (16%)
orf19.7670	-	12 TMs, 1 coiled coil region	plas	-
orf19.988	-	8 TMs	plas	-

1 [35].

2 Nomenclature conflict: the name *DUR3* has been used to refer to orf19.781 and orf19.6656.

Abbreviations: Gtr1 (GTP binding protein Resemblance), Bna4 (Biosynthesis of Nicotinic Acid), Dur31, Dur3 (Degradation of URea), TM (transmembrane domain), RING (Really Interesting New Gene), cyto (cytosol), nucl (nuclear), plas (plasma membrane), mito (mitochondria), cyto (cytosol), ER (endoplasmic reticulum), extr (extracellular), Na^+^-substr-symp (sodium substrate symporter).

**Table 2:** *C. albicans* strains used in this study.

Strain	Genotype	Reference
SC5314	*Candida albicans* wild type	[75]
BWP17	*ura3*::λ*imm434/ura3*::λ *imm434 arg4*::*hisG/arg4*::*hisG his1*::*hisG/his1*::*hisG*	[66]
BWP17+CIp30	*ura3*::λ*imm434/ura3*::λ *imm434 arg4*::*hisG/arg4*::*hisG his1*::*hisG/his1*::*hisG*+CIp30	[9]
*dur31*Δ	*orf19.6656*Δ::*ARG4*/*ORF19.6656*	This study
*dur31*Δ/Δ*ura^-^*	*orf19.6656*Δ::*ARG4*/*orf19.6656*Δ::*HIS1*	This study
*dur31*Δ/Δ	*orf19.6656*Δ::*ARG4*/*orf19.6656*Δ::*HIS1*+CIp10 (*URA3*)	This study
*dur31*Δ/Δ::*DUR31*	*orf19.6656*Δ*::ARG4*/*orf19.6656*Δ*::HIS1*+CIp10 (*ORF19.6656*, *URA3*)	This study
*mkc1*Δ/Δ	*orf19.7523::HIS1/orf19.7523::ARG4*+*pKC70*	[73]
*orf19.1150*Δ	*orf19.1150*Δ::*ARG4*/*ORF19.1150*	This study
*orf19.1150*Δ/Δ	*orf19.1150*Δ::*ARG4*/*orf19.1150*Δ::*HIS1*+CIp10 (*URA3*)	This study
*orf19.1353*Δ	*orf19.1353*Δ::*ARG4*/*ORF19.1353*	This study
*orf19.1353*Δ/Δ	*orf19.1353*Δ::*ARG4*/*orf19.1353*Δ::*HIS1*+CIp10 (*URA3*)	This study
*orf19.2959.1*Δ	*orf19.2959.1*Δ::*ARG4*/*ORF19.2959.1*	This study
*orf19.2959.1*Δ/Δ	*orf19.2959.1*Δ::*ARG4*/*orf19.2959.1*Δ::*HIS1*+CIp10 (*URA3*)	This study
*orf19.3617*Δ	*orf19.3617*Δ::*ARG4*/*ORF19.3617*	This study
*orf19.3617*Δ/Δ	*orf19.3617*Δ::*ARG4*/*orf19.3617*Δ::*HIS1*+CIp10 (*URA3*)	This study
*orf19.3872*Δ	*orf19.3872*Δ::*ARG4*/*ORF19.3872*	This study
*orf19.3872*Δ/Δ	*orf19.3872*Δ::*ARG4*/*orf19.3872*Δ::*HIS1*+CIp10 (*URA3*)	This study
*orf19.5443*Δ	*orf19.5443*Δ::*ARG4*/*ORF19.5443*	This study
*orf19.5443*Δ/Δ	*orf19.5443*Δ::*ARG4*/*orf19.5443*Δ::*HIS1*+CIp10 (*URA3*)	This study
*orf19.5848*Δ	*orf19.5848*Δ::*ARG4*/*ORF19.5848*	This study
*orf19.5848*Δ/Δ	*orf19.5848*Δ::*ARG4*/*orf19.5848*Δ::*HIS1*+CIp10 (*URA3*)	This study
*orf19.6200*Δ	*orf19.6200*Δ::*ARG4*/*ORF19.6200*	This study
*orf19.6200*Δ/Δ	*orf19.6200*Δ::*ARG4*/*orf19.6200*Δ::*HIS1*+CIp10 (*URA3*)	This study
*orf19.6847*Δ	*orf19.6847*Δ::*ARG4*/*ORF19.6847*	This study
*orf19.6847*Δ/Δ	*orf19.6847*Δ::*ARG4*/*orf19.6847*Δ::*HIS1*+CIp10 (*URA3*)	This study
*orf19.7670*Δ	*orf19.7670*Δ::*ARG4*/*ORF19.7670*	This study
*orf19.7670*Δ/Δ	*orf19.7670*Δ::*ARG4*/*orf19.7670*Δ::*HIS1*+CIp10 (*URA3*)	This study
*orf19.988*Δ	*orf19.988*Δ::*ARG4*/*ORF19.988*	This study
*orf19.988*Δ/Δ	*orf19.988*Δ::*ARG4*/*orf19.988*Δ::*HIS1*+CIp10 (*URA3*)	This study

### Screening of a defined set of mutants for reduced oral epithelial cell damage capacity identifies a novel sodium substrate symporter

Oral candidiasis is characterized by *C. albicans* adherence to, invasion into and ultimate damage of oral epithelial cells. We therefore first investigated the capacity of each mutant to damage monolayers of oral epithelial cells *in vitro*.

**Figure 1:**
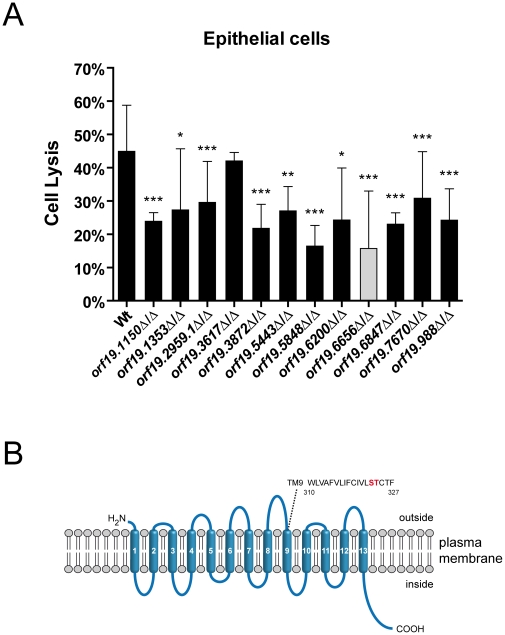
Oral epithelial damage capacity of oral infection-associated *C. albicans* mutants and structural features of a novel sodium/substrate transporter. (A) Oral epithelial cell monolayers were infected with the indicated *C. albicans* wild type (Wt) and mutant strains and incubated for 15 h at 37°C and 5% CO_2_. Host cell damage was determined by measuring lactate dehydrogenase (LDH) levels and expressed as percentage of a total lysis control using 1% Triton X-100. Strain orf19.6656 (gray column) displayed the strongest defect in damage capacity. Results are the mean ± SD of at least two independent experiments, each performed in triplicate. *P<0.05, **P<0.01 and ***P<0.001 compared to the wild type strain. (B) Predicted topological structure of orf19.6656. Transmembrane domains (TM) were predicted using the TMHMM server v. 2.0 and are numbered in white. Extracellular and intracellular loops are shown. A search for amino acids implicated in ion binding in the structurally related *E. coli* PutP transporter identified serine and threonine residues in TM9 (marked in red).

Host cells were infected with the different *C. albicans* strains for 15 hours and the degree of oral epithelial cell damage was quantified by measuring lactate dehydrogenase (LDH) activity [29], [30]. After 15 hours of infection only one mutant (orf19.3617Δ/Δ, lacking a predicted mitochondrial protein with similarity to human Ras-related GTP-binding protein A) caused similar damage as the wild type (Figure 1A). All 11 other mutants were significantly reduced in their capacity to damage these host cells (Figure 1A and Table 1).

Strain orf19.6656Δ/Δ, lacking a predicted plasma membrane localized sodium/substrate transporter with 13 TMs, was most attenuated and caused 65% less epithelial damage in comparison to the wild type. We therefore focused our further investigations on orf19.6656Δ/Δ. Since attenuated damage may be due to reduced adhesion and invasion [12], we tested the epithelial adhesion and invasion capacities of orf19.6656Δ/Δ. Interestingly, adhesion and invasion levels were comparable to the wild type (Figure S1), suggesting stage specific functions at later stages during interaction with epithelial cells.

According to the *Candida* Genome Database (www.candidagenome.org), the gene orf19.6656 has the common name *DUR3*, based on sequence similarities to the *Saccharomyces cerevisiae DUR3* gene. *S. cerevisiae DUR3* encodes a transporter for urea and polyamines [31]–[33]. However, a nomenclature conflict exists for orf19.6656, as the common name *DUR3* has also been used to refer to orf19.781 [34]. We therefore performed alignments using the EMBOSS Needle analysis tool (www.ebi.ac.uk/Tools/psa/emboss_needle) and found the orf19.781 predicted protein sequence to be 52.5% identical and 68.5% similar to *S. cerevisiae* Dur3 while orf19.6656 encodes a predicted protein with only 14.7% identity and 29.9% similarity with ScDur3. Therefore, in comparison to orf19.781, orf19.6656 is only distantly related to *ScDUR3*.

We performed a BLASTp analysis of the orf19.6656 protein sequence and found the highest sequence similarities for proteins of unknown function in *Candida dubliniensis* (*CD36_53230*, 95.6% identity, 98.3% similarity) and *Candida tropicalis* (*CTRG_05438*, 80.1% identity, 88.2% similarity). Interestingly, orf19.6656 is 81.2% identical and 90.5% similar to *Pichia stipitis DUR8*. However, *DUR8* has not yet been functionally characterized in *P. stipitis*. Analysis with the SMART (www.smart.embl-heidelberg.de), ExPASy (www.expasy.ch/prosite) and Wolf PSORT (www.wolfpsort.org) databases suggested that *C. albicans* orf19.6656 encodes a plasma membrane localized sodium substrate-symporter of the sodium substrate symporter family (SSSF) containing 13 transmembrane (TM) domains (Figure 1B, Table 1). We furthermore identified amino acids in TM9 which have been shown to be critical for ion binding in *E. coli* PutP, a sodium proline symporter belonging to the SSSF. Based on a recent publication [35], we refer to the *C. albicans* gene orf19.6656 as *DUR31* (degradation of urea). For further analysis, we next constructed a *dur31*Δ/Δ::*DUR31* complemented strain.

### Deletion of *DUR31* affects cell wall integrity in *C. albicans*

**Figure 2:**
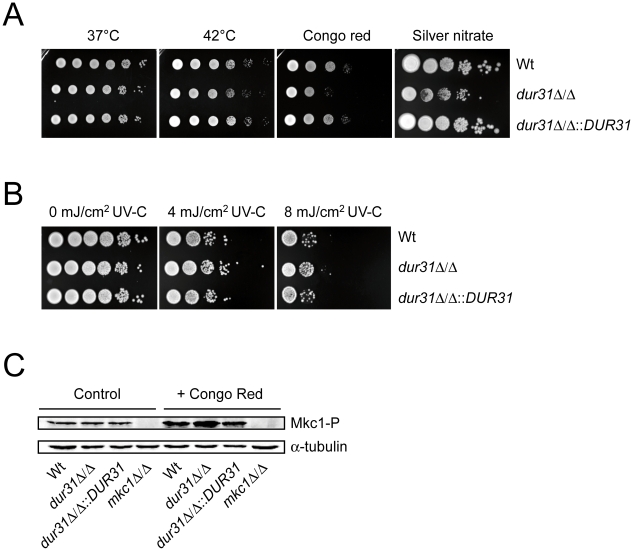
Dur31 contributes to cell wall integrity. Drop test analysis with serial dilutions of *C. albicans* wild type (Wt), *dur31*Δ/Δ mutant and *dur31*Δ/Δ::*DUR31* complemented mutant strain ranging from 10^6^ to 10^1^ cells (left to right) on agar containing or exposed to different stressors. (A) Growth of the *dur31*Δ/Δ mutant on SD agar under thermal stress (42°C), cell wall stress (450 µg ml^−1^ Congo red) and heavy metal stress (0.75 mM silver nitrate). Plates subjected to thermal stress were incubated for 4–5 days and cells grown under cell wall or reductive stress for 2–3 days at 37°C. (B) The *dur31*Δ/Δ mutant displays moderately enhanced tolerance towards UV stress. Serial ten-fold dilutions were prepared and spotted onto YPD agar plates. Cells were then exposed to the indicated intensities of UV-C light and subsequently incubated at 37°C for 2 days. (C) Western blot analysis for identification of phosphorylated Mkc1. *dur31*Δ/Δ has increased levels of phosphorylated Mkc1 upon treatment with the cell wall attacking agent Congo red. The wild type (Wt), *dur31*Δ/Δ mutant, *dur31*Δ/Δ::*DUR31* complemented strain and *mkc1*Δ/Δ mutant were grown under non-stress conditions (control) or conditions of cell wall stress (+Congo red) for 4 hours at 30°C. Protein extracts were blotted and probed for phosphorylated Mkc1 (Mkc1-P). Blots were then stripped and re-probed for α-tubulin (loading control). The *mkc1*Δ/Δ mutant was included as a negative control for the Mkc1-P detection.

Due to the predicted localization of Dur31 in the plasma membrane (Table 1 and Figure 1), we investigated the effect of different cell wall and plasma membrane disturbing agents and stresses on growth of the *dur31*Δ/Δ mutant. Deletion of *DUR31* did not affect growth of *C. albicans* on SD agar at 37°C and 42°C (Figure 2A). However, the *dur31*Δ/Δ mutant was more sensitive to cell wall stress induced by 450 µg ml^−1^ Congo red. Complementation of the mutant with *DUR31* restored growth under these stress conditions. Western blot analysis of strains grown under conditions of cell wall stress revealed higher levels of phosphorylated Mkc1, a marker for cell wall perturbances [36], in the *dur31*Δ/Δ mutant in comparison to the wild type and complemented strains (Figure 2C). Heavy metal stress, induced by 0.75 mM silver nitrate, led to 100- to 1000-fold reduction in growth of the *dur31*Δ/Δ mutant in comparison to the wild type and *dur31*Δ/Δ::*DUR31* complemented strain. Osmotic stress (1.5 M NaCl) had no effect on growth of the mutant (data not shown). The *dur31*Δ/Δ mutant was moderately more tolerant to UV(-C)-stress than the wild type and complemented strain (Figure 2B). We furthermore investigated the effect of the membrane potential-disrupting ionophore nigericin (Fluka) on growth of the *dur31*Δ/Δ mutant but did not detect a difference in susceptibility in comparison to the wild type (data not shown). Collectively, these results indicate that deletion of *DUR31* affects cell wall integrity in *C. albicans*.

### A *dur31*Δ/Δ mutant displays defective colony and microcolony formation

Amongst the range of putative SSSF substrates, polyamines and certain amino acids such as arginine have been demonstrated to induce filamentation [37], [38]. Since the production of filaments is one of the major virulence traits of *C. albicans* [39], we next investigated the morphology of *dur31*Δ/Δ under various hypha-inducing conditions.

**Figure 3:**
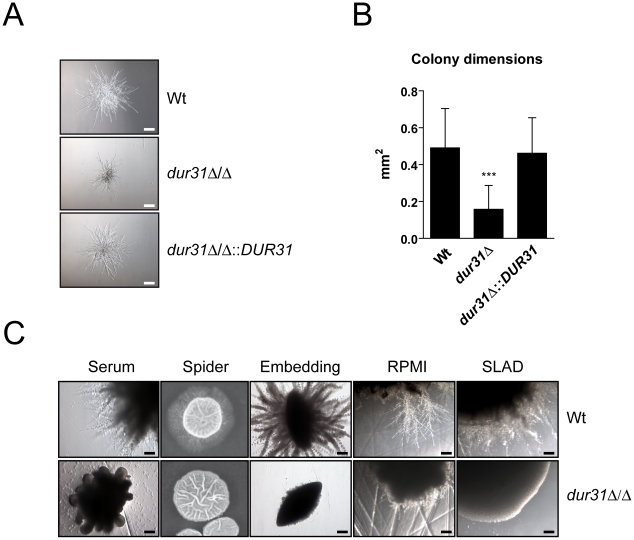
Dur31 is required for filamentous colony formation. (A) *dur31*Δ/Δ displays defective microcolony formation. For induction of hyphal microcolonies, fungal cells were grown overnight to stationary phase in YPD medium, washed twice with PBS, and resuspended in PBS. 100 cells per strain were inoculated into 500 µl RPMI medium per well of a 24-well cell culture plate and incubated at 37°C for 24 hours in presence of 5% CO_2_. Representative pictures are shown. Scale bar: 100 µm. (B) Quantification of wild type (Wt), *dur31*Δ/Δ mutant and *dur31*Δ/Δ::*DUR31* revertant microcolony dimensions after incubation of fungal cells in RPMI medium at 37°C for 24 hours in presence of 5% CO_2_. Results are the mean ± SD of two independent experiments, each performed in quadruplicate. ***P<0.001 compared with the wild type and *dur31*Δ/Δ::*DUR31* complemented strain. (C) Analysis of *C. albicans* wild type (Wt) and *dur31*Δ/Δ mutant filamentation by plating 50 cells per strain on solid water agar supplemented with either 10% fetal bovine serum or 5% RPMI, or by plating cells on solid Spider or SLAD medium. For embedding approximately 50 cells per strain were added to molten YPS (YP-saccharose) agar. The agar was allowed to solidify and plates were subsequently incubated for 5 days at 25°C. RPMI agar and SLAD agar plates were incubated for 4 days, serum agar plates for 2 days, and Spider agar plates for 10 days at 37°C. Experiments were performed twice in duplicate. Representative pictures are shown. Scale bar: 100 µm.

We first analyzed filamentation of *dur31*Δ/Δ on a single cell level on a plastic surface in liquid hyphae inducing media (RPMI or 10% serum, Figure S2A). By 3 h, the *dur31*Δ/Δ mutant formed filaments of similar length and morphology to the wild type, indicating that *DUR31* is dispensable for the early stages of hyphal formation in response to liquid serum or RPMI. Early stage (3 h) hyphal formation on human-derived oral epithelial cell monolayers was also comparable to that of the wild type (Figure S2B). However, when we extended the incubation time from 3 to 24 h, the *dur31*Δ/Δ mutant formed significantly smaller microcolonies than the wild type and complemented strains (Figure 3A and 3B). Therefore, although dispensable for initial hyphal growth, *DUR31* appears to be required for further filamentation and the production of regular microcolonies.

We therefore next grew *dur31*Δ/Δ on agar containing 10% serum or 5% RPMI, on Spider or SLAD agar or by embedding in YPS agar. Under each condition tested, the *dur31*Δ/Δ mutant formed aberrant colonies which lacked the peripheral, invasive filaments observed for the wild type (Figure 3C).

Together our data demonstrate that although *DUR31* is not required for the initiation of hyphal formation in liquid media, this gene is required for the continued development of multicellular filamentous structures such as colonies.

### Models of systemic infection: *DUR31* is required for endothelial damage and immune evasion

*DUR31* was exclusively upregulated during oral infection, and not during blood and liver infection [9], [23], [40]. However, the stress resistance- and hyphal formation- defects, together with the strong attenuation in epithelial damage, led us to postulate that Dur31 may also function during other forms of candidiasis.

**Figure 4:**
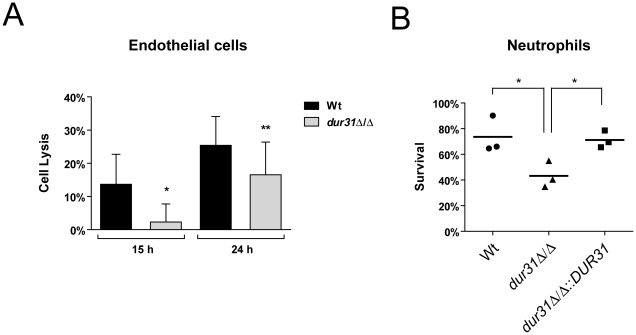
Dur31 is required for damage of human endothelial cells and for immune evasion. (A) Monolayers of HUVEC endothelial cells were infected with *C. albicans* wild type (Wt) and *dur31*Δ/Δ mutant strains for 15 or 24 h. Damage of host cells was determined by measuring lactate dehydrogenase (LDH) levels. Results are the mean ± SD of at least three independent experiments, each performed in triplicate. *P<0.05 and **P<0.01 compared to the wild type strain. (B) A *dur31*Δ/Δ mutant is more susceptible to killing by human neutrophils. Wild type (Wt), *dur31*Δ/Δ mutant and *dur31*Δ/Δ::*DUR31* complemented mutant cells were exposed to human neutrophils for three hours and viability was determined by plating on YPD agar. Experiments were performed three times. The result values are indicated by circles, triangles or rectangles. The bar represents the mean of these single values. *P<0.01 compared with the wild type and *dur31*Δ/Δ::*DUR31* complemented strain.

During systemic infections, *C. albicans* must adhere to and traverse the endothelial lining of blood vessels in order to access internal organs. Moreover, *C. albicans* must resist attacks by the immune system. We therefore next investigated whether *DUR31* is required for damage of endothelial cells and tolerance to killing activities by human neutrophils (Figure 4). We used monolayers of HUVEC endothelial cells for infection with the different *C. albicans* strains *in vitro*. Following a 15 or 24 hour co-incubation we determined host cell damage by measuring LDH release. The *dur31*Δ/Δ mutant caused significantly reduced damage to endothelial cells after 15 and 24 hours of infection (Figure 4A). The mutant caused 83% less damage in comparison to the wild type after 15 hours and 35% less damage after 24 hours post infection. These results indicate that Dur31 is not only required for damage of epithelial cells, but also for endothelial cell damage and therefore may play a role during systemic infections.

Neutrophils play a key role in host defense against *C. albicans* infections [23], [41]. We therefore investigated survival of the *dur31*Δ/Δ mutant in the presence of these phagocytes. While 73.6% of wild type cells survived exposure to neutrophils, only 43.2% of *dur31*Δ/Δ cells remained viable following co-incubation with these phagocytes (Figure 4B). Complementation with *DUR31* restored wild type (71.2%) survival rates. The generation of reactive oxygen species (ROS) plays an important role in neutrophil-killing of microbes [41], [42]. We therefore investigated whether the higher susceptibility of the *dur31*Δ/Δ mutant to neutrophils was due to increased oxidative stress sensitivity. However, using a spot dilution assay with SD medium containing 2 mM H_2_O_2_ or 0.4 mM menadione, no growth defect was observed for the mutant in comparison to the wild type (data not shown). Alongside neutrophils, macrophages also play an important role in host defense. We thus tested survival of the mutant in the presence of macrophages. Here, although a trend towards reduced survival of the *dur31*Δ/Δ mutant was observed, the difference was not significant (data not shown).

The reduced endothelial damage capacity of *dur31*Δ/Δ together with reduced survival in the presence of neutrophils strongly implied that *DUR31* may play a role during systemic candidiasis.

### Deletion of *DUR31* attenuates virulence in a mouse model of hematogenously disseminated candidiasis

**Figure 5:**
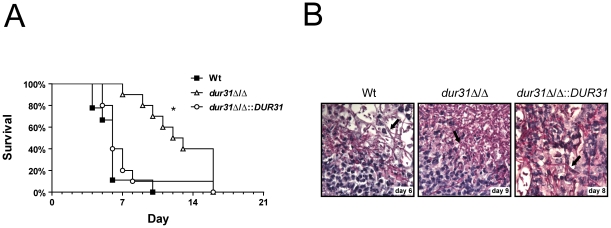
*DUR31* deletion attenuated virulence in a mouse model of hematogenously disseminated candidiasis. (A) Female Balb/C mice (n = 10 mice per *C. albicans* strain) were challenged intravenously with either the wild type (Wt), the *dur31*Δ/Δ mutant or the *dur31*Δ/Δ::*DUR31* complemented strain via the lateral tail vein and survival followed for up to 21 days. *P<0.0001 compared with mice either infected with the wild type or *dur31*Δ/Δ::*DUR31* complemented strain. (B) Periodic acid Schiff staining of kidney sections from mice infected with the wild type, the *dur31*Δ/Δ mutant or the *dur31*Δ/Δ::*DUR31* complemented strain at the indicated time points post infection. Pictures were taken at 63× magnification. Arrows point to *C. albicans* filaments within the tissue.

We determined the virulence of the *dur31*Δ/Δ mutant using a murine model of hematogenously disseminated candidiasis (Figure 5). Survival of mice infected with the wild type or the *dur31*Δ/Δ::*DUR31* complemented strain showed no significant difference (Figure 5A). The majority of mice infected with these strains died within 5 to 8 days post infection (p.i.). In contrast, all mice infected with the *dur31*Δ/Δ mutant strain were still alive at day 6 p.i.. Starting at day 7 p.i., however, mice began to succumb to infection and died between day 7 and day 13 p.i.. Therefore, *DUR31* is required for the rapid onset of symptomatic disseminated candidiasis.

To investigate whether the *dur31*Δ/Δ mutant also exhibited morphological defects *in vivo*, we performed histological analysis of the kidneys of end-point mice. Interestingly, despite delayed mortality of *dur31*Δ/Δ-infected mice, upon death, the kidneys exhibited similar pathologies to wild type-infected mice with the presence of fungal filaments and neutrophil infiltrates (Figure 5B).

Given the importance of Dur31 for different stages of pathogenesis and its proposed role as a transporter, we next sought to identify its substrate(s).

### Dur31 facilitates spermidine utilization and histatin 5 susceptibility

**Figure 6:**
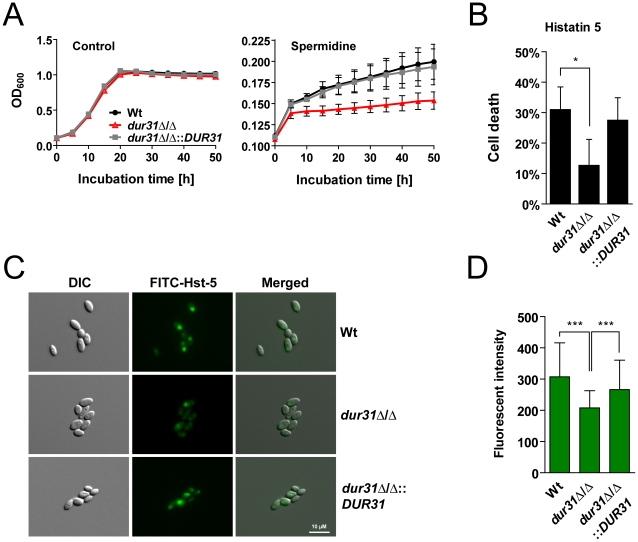
Dur31 mediates both spermidine assimilation and histatin 5 sensitivity. (A) Growth curve for the wild type (Wt), the *dur31*Δ/Δ mutant and the *dur31*Δ/Δ::*DUR31* complemented strain with spermidine as sole carbon source. Fungal overnight SD cultures were washed once in water and adjusted to an OD_600_ of 0.1 in yeast nitrogen base (YNB) medium supplemented with 100 µg ml^−1^ spermidine. Growth was monitored in an ELISA reader at 37°C for 50 h. Results are the mean ± SEM of two independent experiments, each performed in triplicate. (B) A *dur31*Δ/Δ mutant is more tolerant towards histatin 5. Fungal overnight YPD cultures were washed twice in water and adjusted to 10^6^ cells ml^−1^ in 10 mM NaPB. Cells were then incubated with 15 µM histatin 5 for 1 h at 30°C and shaking (300 rpm). Dilutions were plated on YPD agar plates and incubated at 30°C for 2 d for determination of colony forming units. The percentage cell death was determined compared with untreated cells. Results are the mean ± SD of three independent experiments, each performed in duplicate. *P≤0.05 compared with the wild type (Wt). (C) Differential interference contrast (DIC) and fluorescent microscopic images showing uptake of FITC-histatin 5 (30 µM) by wild type (Wt), *dur31*Δ/Δ mutant and *dur31*Δ/Δ::*DUR31* complemented cells after 15 min co-incubation at 30°C. The *dur31*Δ/Δ mutant translocates FITC-histatin 5 less efficiently than the Wt and revertant. Scale bar: 10 µM. (D) Quantification of the FITC-histatin 5 mean fluorescent intensity of at least 80 cells per strain. Results are the mean ± SD of two independent experiments. ***P<0.001 compared with the wild type and *dur31*Δ/Δ::*DUR31* complemented strain.

Members of the sodium substrate symporter family have been shown to transport a variety of different substrates, including urea, sugars, amino acids, polyamines, vitamins, ions and water [43]. Because *DUR31* was originally annotated as a urea transporter (*DUR3*, above) we investigated growth of the *dur31*Δ/Δ mutant on urea as sole carbon and nitrogen source, but did not find a difference in growth in comparison to the wild type (data not shown). Additionally, transcriptional data from a recent publication [34] support the view that Dur31 probably does not transport urea. We therefore investigated growth of the *dur31*Δ/Δ mutant in the presence of different sugars and amino acids as sole carbon or nitrogen sources, but again did not find differences in growth between the mutant and the wild type (data not shown). When we investigated growth of the mutant with the polyamine spermidine as sole carbon source, only moderate growth of the wild type and complemented strains was observed (Figure 6A), however the *dur31*Δ/Δ mutant did not grow at all, indicating that Dur31 might transport this polyamine or a related amine-containing compound. Indeed, in a parallel study, the laboratory of Mira Edgerton demonstrated that Dur3, and to a lesser extent also Dur31, function as spermidine transporters [35]. These results are in agreement with our own data that Dur31 facilitates spermidine import.

One of the key mechanisms which prevent oral *C. albicans* infections in healthy individuals is the production of antimicrobial peptides such as the histidine-rich histatins. Among the histatins, salivary histatin 5 is the most effective candidacidal peptide [44]. Interestingly, HIV patients often exhibit lower levels of histatin 5 [45]. Since *DUR31* was specifically upregulated in *C. albicans* samples from HIV patients with oral candidiasis (low histatin 5), we postulated that Dur31 might function in the absence of histatin 5 *in vivo*. Strikingly, the mutant was significantly more resistant to killing by this antimicrobial peptide in comparison to the susceptible wild type and the *dur31*Δ/Δ::*DUR31* complemented strain (Figure 6B). We used FITC-labeled histatin 5 to monitor uptake by the *dur31*Δ/Δ mutant (Figure 6C). Cells were incubated for 15 min at 30°C with 30 µM FITC-histatin 5 and immediately visualized with fluorescence microscopy. FITC-histatin 5 was detected intracellularly, as previously described [46]. However, in comparison to the wild type and complemented strain, fewer mutant cells had taken up FITC-histatin 5. We quantified the fluorescence intensities of approximately 100 cells of each strain and found the *dur31*Δ/Δ mutant to have 32% reduced levels of FITC-histatin 5 in comparison to the wild type (Figure 6D). Therefore the increased survival of the *dur31*Δ/Δ mutant in the presence of histatin 5 correlates with decreased internalization of this antimicrobial peptide.

Histatin 5 binding to the *C. albicans* cell surface is mediated by Ssa1 and Ssa2 [47], [48]. To ensure that the reduced histatin 5-uptake and increased survival of the *dur31*Δ/Δ mutant was not due to altered levels of Ssa1/2 on the cell surface, we performed indirect immunofluorescence with a mouse anti-Hsp70 monoclonal antibody, which detects Ssa1/Ssa2 (Figure S3A). The *dur31*Δ/Δ mutant had comparable levels of Hsp70 proteins on the cell surface (Figure S3B). In addition, we performed western blotting with the anti-Hsp70 antibody on cell wall extracts of the wild type and *dur31*Δ/Δ mutant. Comparable levels of cell surface-associated Ssa1/2 were detected in both the wild type and *dur31*Δ/Δ mutant (Figure S3C). These data are in agreement with the recent report of Kumar et al. [35], who also provided evidence that Dur31 is a histatin 5 transporter.

Together, these results indicate that Dur31 is likely to transport both, the polyamine spermidine and the antimicrobial peptide histatin 5.

### Dur31 mediates extracellular alkalinization

In a recent publication it has been elegantly demonstrated that *C. albicans* is able to actively alkalinize its environment, thereby auto-inducing hyphal formation [17]. In the current study we have shown that the *dur31*Δ/Δ mutant is defective for hyphal formation and that *DUR31* encodes a predicted sodium substrate transporter. Given its proposed role in transporting amine-containing molecules (necessary for environmental alkalinization/hyphal auto-induction [17]), we hypothesized that Dur31 may mediate extracellular alkalinization, thereby mediating hyphal auto-induction.

**Figure 7:**
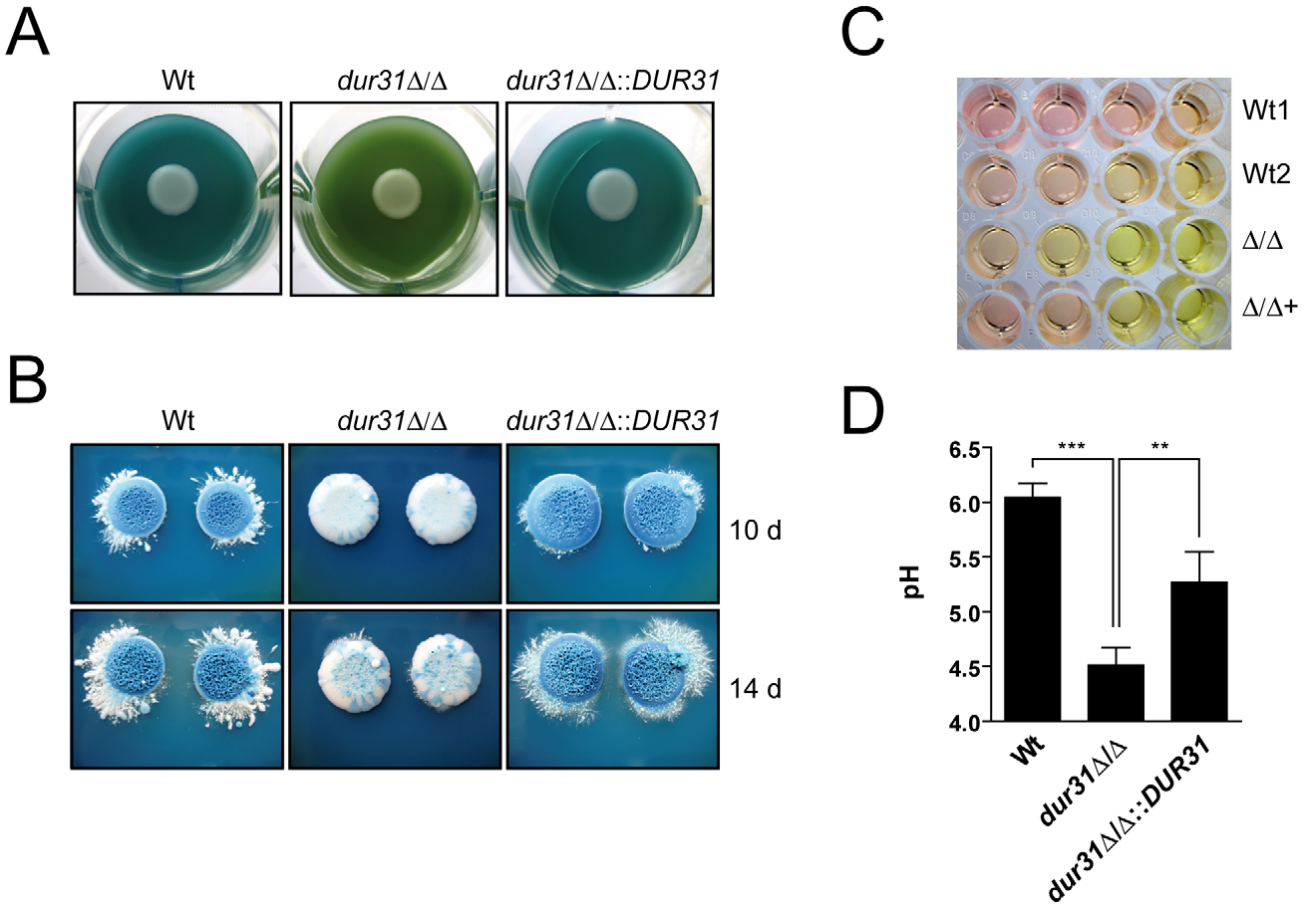
Dur31 mediates extracellular alkalinization. (A and B) *dur31*Δ/Δ mutant colonies on GM-BCG agar (pH 4) display defective regulation of extracellular pH. *C. albicans* strains were grown overnight in YPD, washed once in water and diluted to an OD_600_ of 1.0 in water. Seven µl of this dilution were pipetted onto the agar, with one strain per well. 12-well plates were incubated at 30°C for four days (A), and 6-well plates were incubated for 10 or 14 days (B). A shift from green to blue indicates alkalinization. The *dur31*Δ/Δ mutant colonies failed to alkalinize the medium to the same extent as the wild type. (A) The extracellular medium remained green, indicating that the pH did not significantly increase. In contrast, extracellular medium of the wild type and the revertant strain turned blue, indicating a shift in pH from 4 to >5.4. Experiments were performed in quadruplicate and repeated three times. Representative pictures are shown. (B) Following extensive incubation times (10 or 14 days, B), *dur31*Δ/Δ finally alkalinized the surrounding medium. By this time point, wild type and complemented strains formed blue colonies. Experiments were performed on three different occasions. Representative pictures are shown. (C) A *dur31*Δ/Δ mutant is defective in alkalinization of medium 199. Strains SC5314 (Wt1), BWP17+CIp30 (Wt2), the *dur31*Δ/Δ mutant (Δ/Δ) and the *dur31*Δ/Δ::*DUR31* complemented strain (Δ/Δ+) were inoculated at an OD_600_ of 1.0 in medium 199, pH 4 in a 96-well plate. Serial five-fold dilutions were prepared and plates were incubated at 37°C for 24 h. Alkalinization of the medium is indicated by a change of colour from yellow to red. Experiments were performed in duplicate on three different occasions. Representative pictures are shown. (D) Quantification of the *dur31*Δ/Δ alkalinization defect. YPD overnight cultures of the wild type (Wt), *dur31*Δ/Δ mutant and *dur31*Δ/Δ::*DUR31* complemented strain were adjusted to OD 0.1 in medium 199 (pH 4) and grown for 24 hours at 37°C. Results are the mean ± SD of two independent experiments, each performed in duplicate. **P<0.01 and ***P<0.001.

We used two approaches for visualization of extracellular alkalinization: solid GM medium supplemented with bromocresol green as pH indicator and liquid medium 199 with phenol red as pH indicator. Both media were initially adjusted with HCl to pH 4. The wild type and *dur31*Δ/Δ::*DUR31* complemented strain alkalinized solid GM-BCG medium within three to four days, indicated by a change of medium colour from green to blue (Figure 7A). This colour change indicates a shift in pH from 4 to >5.4. In contrast, the *dur31*Δ/Δ mutant did not alkalinize the medium as efficiently. After three days of incubation, the medium colour surrounding *dur31*Δ/Δ colonies remained green, indicating that the extracellular medium was still in the range of pH 4 at that time. Following more extensive incubation times (10 and 14 days), *dur31*Δ/Δ was eventually capable of alkalinizing the surrounding medium (Figure 7B); however, by this time point, the wild type and *dur31*Δ/Δ::*DUR31* colonies formed a strong blue, whilst *dur31*Δ/Δ colonies remained white.

In liquid medium 199, alkalinization is indicated by a colour change from yellow (pH 4) to red (pH 6–7). Similar to the results obtained on solid GM-BCG medium, the *dur31*Δ/Δ mutant again displayed defective alkalinization (Figure 7C). To confirm this observation, we adjusted medium 199 to pH 4, grew the strains for 24 h and directly measured alkalinization with a pH meter. Within this time, wild type *C. albicans* raised the medium pH from 4 to pH 6 (Figure 7D). In contrast, the *dur31*Δ/Δ mutant alkalinized the surrounding medium to only pH 4.5. Complementation of the mutant with a single copy of *DUR31* significantly increased medium alkalinization. Together these data demonstrate that Dur31 is involved in extracellular alkalinization.

## Discussion

### Novel oral infection-associated *C. albicans* genes

Of the 6203 predicted *C. albicans* open reading frames (ORFs), around 75% (4672 ORFs) are still uncharacterized (*Candida* Genome Database) [49]. The high number of uncharacterized genes within the genome of *C. albicans* makes it highly likely that important fungal virulence factors have yet to be discovered [50]. We therefore characterized the role of 12 novel *C. albicans* genes that we predicted to be involved in oral infection. These genes were selected based on their expression during oral infection [9] and *in silico* predicted structural/functional features. Strikingly, 11 mutants were significantly reduced in their capacity to damage oral epithelium, suggesting effective selection criteria. We thereafter focused our analysis on orf19.6656 (*DUR31*) as a mutant lacking this gene had the strongest defect in damage. In order to cause epithelial damage, *C. albicans* first has to adhere to and subsequently invade into host cells [9], [12], [13], [30], [51]–[53]. Despite the strong reduction in epithelial damage observed upon *DUR31* deletion, the mutant adhered to and invaded epithelial cells at levels comparable to the wild type. This is most likely the result of wild type rates of germ tube formation by *dur31*Δ/Δ as initial hyphal formation is generally sufficient to allow epithelial adhesion and invasion [12]. Consequently, the attenuation in epithelial damage must be due to other fungal activities (see below).

### *DUR31* mediates multiple stages of candidiasis

*DUR31* was observed to be exclusively upregulated by *C. albicans* during clinical oral candidiasis and was required for oral epithelial damage. However, we found *DUR31* to be involved in multiple interactions associated with systemic candidiasis and *dur31*Δ/Δ exhibited significantly reduced virulence *in vivo*. If *C. albicans* gains access to the bloodstream it can infect virtually every internal organ and kill the host. First, however, the fungus must survive the hostile milieu of the blood and then traverse the endothelial lining of blood vessels. Within the blood, neutrophils are believed to kill *C. albicans* via a combination of acute nutrient starvation, oxidative stress and the action of antimicrobial peptides. Although *dur31*Δ/Δ grew normally in the presence of reactive oxygen species, increased sensitivity to cell wall stress may partially account for the higher killing rate of *dur31*Δ/Δ compared to the wild type. Interestingly, the neutrophil-associated antimicrobial peptide, defensin-1 kills *C. albicans* via a pathway related to histatin 5-mediated killing [54]. As Dur31 likely mediates histatin 5 import (below), it is possible that deletion of *DUR31* also altered sensitivity to other antimicrobial peptides, which are present in neutrophils.

*C. albicans* cells which survive attack by blood components can next penetrate the endothelial lining of blood vessels to access other organs. During traversal of the endothelial cell layer, the fungus damages these host cells [51], [55]. Therefore, the reduced endothelial damage potential of *dur31*Δ/Δ may reflect an impaired capacity to disseminate through blood vessels during systemic candidiasis. Indeed, *C. albicans* mutants with reduced capacity to damage endothelial cells *in vitro*, often display reduced virulence in mice models of hematogenously disseminated candidiasis [56].

Deletion of *DUR31* also resulted in aberrant filamentous growth *in vitro*, a phenotype often associated with reduced virulence [16], [29], [51]. However, histological examination of the kidneys of endpoint *dur31*Δ/Δ-infected mice revealed filamentous fungal foci comparable to the wild type. It would appear, therefore, that deletion of *DUR31* delayed the maturation of larger fungal lesions, rather than preventing their formation completely. This view fits with the eventual fate of *dur31*Δ/Δ-infected mice: delayed, yet ultimately complete mortality. Therefore, Dur31 is involved in the rapid onset of symptomatic systemic candidiasis but not its final conclusion. However, in the hospital setting, following the onset of hypotension, each hour of delay in starting antifungal treatment increases the mortality of candidaemia by around 5% [24]. Therefore, the contribution of Dur31 to virulence may be clinically relevant.

In summary, a combination of increased sensitivity to neutrophils, together with the reduced endothelial damage capacity and morphological defects of *dur31*Δ/Δ cells, likely contributes to the delayed virulence of this strain.

### Dur31 mediates polyamine utilization, histatin 5-sensitivity and extracellular alkalinization

*In silico* analysis suggested that *DUR31* encodes a plasma membrane localized protein with 13 transmembrane domains, belonging to the sodium/substrate symporter family (SSSF). Indeed, the common topological motif of SSSF proteins has been defined as an arrangement of 13 transmembrane domains with an extracellular N-terminus and cytoplasmic C-terminus [43]. Each of these topological features was present in the Dur31 sequence. Moreover we identified the amino acids serine and threonine in the transmembrane domain TM9 of *C. albicans* Dur31, a feature which has been shown to be critical for ion binding in *Escherichia coli* PutP, a sodium proline symporter of the SSSF [43]. Therefore, *in silico* analysis suggests that *DUR31* encodes a sodium substrate transporter.

Members of the SSSF transport a wide range of substrates, including sugars, amino acids (e.g. proline), vitamins (e.g. pantothenate), ions, urea and water [43]. As these substrates are often transported against a concentration gradient, cells use a sodium motive force to fuel substrate uptake. The sodium motive force is built up by sodium pumps or sodium/proton antiporters. Most of the SSSF transporters are involved in the acquisition of their respective substrate as carbon or nitrogen sources [43]. Interestingly, the *Staphylococcus aureus* orthologue of *E. coli* PutP is a virulence factor: a *S. aureus putP* mutant had approximately 10-fold reduced virulence in wound and murine abscess infection models compared to a wild type control [57]. Based on these data and the fact that *C. albicans* Dur31 was required for normal virulence in a murine infection model, we reasoned that *DUR31* might encode a sodium/proline symporter; however, we did not find evidence of Dur31-mediated proline uptake by *C. albicans* (data not shown). Based on apparent sequence similarities to *S. cerevisiae DUR3*, *C. albicans DUR31* (orf19.6656) was originally designated the common name, *DUR3* (CGD). However, orf19.781 is also named *DUR3*. In *S. cerevisiae*, *DUR3* encodes a urea and polyamine transporter. A recent study demonstrated that orf19.781 is the major urea transporter in *C. albicans* [34]. This finding was supported by our *in silico* analyses which indicated that orf19.781, and not orf19.6656, is the true orthologue of *ScDUR3*. Indeed phylogenetic analysis revealed that *ScDUR3* and *CaDUR3* (orf19.781) cluster together with Dur3 orthologues from other fungal species such as *Aspergillus fumigatus*, *Aspergillus terreus*, *Magnaporthe oryzae*, *Schizosaccharomyces pombe* and *Cryptococcus gattii* (Figure S4). CaDur31 (orf19.6656) on the other hand, belonged to an independent cluster with proteins of unknown function from species of the CUG-clade and non-CUG-clade fungal species including *Candida glabrata*, *Kluyveromyces lactis*, *Coccidioides immitis*, *Neurospora crassa*, *Malassezia globosa* and *Ustilago maydis*. No *DUR31* orthologues were detected in *S. cerevisiae* or *A. fumigatus*. We therefore propose that *C. albicans DUR31* has evolved separately or diverged from *DUR3* and is present in a limited yet diverse range of fungal species, including many pathogenic species. Therefore, together with the results of Navarathna et al. [34], we conclude that *DUR31* is unlikely to encode a urea transporter.

In order to determine which substrate or substrate family Dur31 might transport, we systematically analyzed the growth of the *dur31*Δ/Δ mutant in the presence of known SSSF-substrates as sole C- or N-source. The mutant displayed no growth defects with sugars, amino acids or the vitamin pantothenate as nutrient sources (data not shown). Importantly however, *dur31*Δ/Δ failed to utilize the polyamine spermidine as sole C-source. This indicates that Dur31 may transport the polyamine spermidine. Polyamines are essential for cell growth and for modulating the function of nucleic acids and ATP [58]. In *S. cerevisiae*, polyamine uptake is mainly catalyzed by Dur3 and Sam3 [33]. In *C. albicans*, the role of Dur31, and another transporter, Dur3, in polyamine transport has been independently investigated by the laboratory of Mira Edgerton [35]. These authors demonstrated that Dur3 and Dur31 share overlapping functions in transporting spermidine in *C. albicans*. Interestingly, polyamines have also been implicated in filament formation [37], providing a potential link between defective polyamine transport in the *dur31*Δ/Δ mutant and the resulting filament formation defect on semi-solid media.

**Figure 8:**
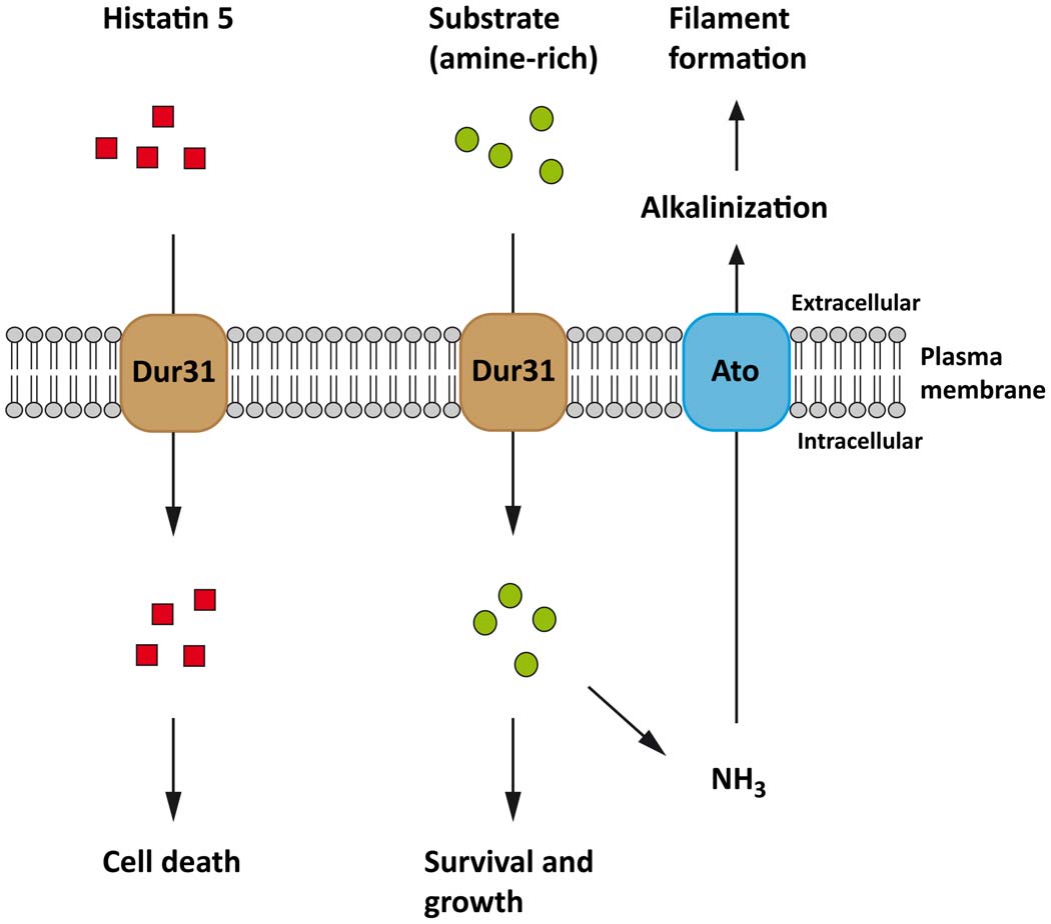
Proposed model for *C. albicans* Dur31. Dur31 functions as a transporter of both amine-containing substrates and the antimicrobial peptide histatin 5 in *C. albicans*. Histatin 5 (red rectangles) is transported into the cell by Dur31 which leads to cell death. Dur31 also transports an amine-containing substrate (green circles) which is beneficial for the cell, as it promotes growth, alkalinization of the extracellular environment through amine exporters (Ato) and concomitant filament formation required for full virulence. The Ato transporter has been included into the model based on [17].

Therefore, expression of *DUR31* likely benefits *C. albicans* by allowing the fungus to utilize distinct amine-containing substrates and facilitating hyphal growth. Interestingly, we also found a role for Dur31 that may benefit the infected host, rather than the fungus itself. The cationic antimicrobial peptide histatin 5 protects the host from microbial infections of the oral cavity and has been shown to efficiently kill *C. albicans* [59]. Opposed to other cationic antimicrobial peptides, histatin 5 does not induce disruption of the fungal cell membrane but rather exerts its killing activity intracellularly. Therefore, the transport of histatin 5 into the cell has been defined as an essential process for its antifungal activity [59]. The laboratory of Mira Edgerton has demonstrated that the two cell wall localized heat shock protein 70 family members Ssa1 and Ssa2 bind histatin 5 [47], [48]. However, the mechanism of internalization has, until very recently, remained unknown [35], [60]. We initially investigated susceptibility of *dur31*Δ/Δ to killing by histatin 5 based on the specific transcriptional upregulation of *DUR31* in samples from HIV^+^/AIDS patients with oral candidiasis – patients in which histatin 5 levels are much lower than in healthy individuals [45], [61]. Indeed, we found that Dur31 mediates killing of *C. albicans* by histatin 5. Similarly, deletion of the histatin 5 receptor encoding gene (*SSA2*) also led to enhanced *C. albicans* survival in the presence of this antimicrobial peptide, with *ssa2*Δ/Δ survival values comparable to that of the *dur31*Δ/Δ mutant [59]. Together with the work of the Edgerton laboratory, our data support a model whereby histatin 5 binds Ssa1/2 on the surface of *C. albicans* and is then transported, via Dur31, into the cell, subsequently killing the fungus (Figure 8). The interaction of histatin 5 with Dur31 likely represents an example of co-evolution, whereby the fungus expresses a transporter to acquire a nutrient – in this case polyamines – and the host expresses a cytotoxic substrate for this very transporter. The fact that *DUR31* is expressed by *C. albicans* infecting HIV^+^ patients (where histatin 5 expression is impaired) supports this view. In the future, it will be intriguing to investigate *DUR31* expression levels by *C. albicans* colonizing the oral cavity of humans with normal levels of histatin 5 expression. Moreover, elucidating the molecular mechanism of histatin 5 import by Dur31 will provide invaluable insight into how the host immune system “tricks” microorganisms into taking up this highly cytotoxic compound.

Finally we provide a mechanism of Dur31-mediated filamentation. It has recently been shown that *C. albicans* actively alkalinizes its surrounding environment, thereby auto-inducing filamentation [17]. Indeed, these authors propose that nutrient-starved *C. albicans* cells import and catabolize amino acids, whereby the amine groups are removed during substrate entry to the tricarboxylic acid cycle, converted to volatile ammonia and excreted, thereby raising the environment pH. As Dur31 was required for polyamine utilization, extracellular alkalinization and hyphal morphogenesis, we propose that this transporter is involved in the import of amine-containing substrates (such as spermidine), which feed into the hyphal auto-induction pathway described by Vylkova et al. [17] (Figure 8). These authors identified 10 *C. albicans* proteins encoded by the *ATO* (ammonia transport outward, [62]) family, of which Ato5 appears to be critical for extracellular alkalinization, as deletion of *ATO5* renders *C. albicans* cells unable to neutralize their environment [17]. We propose that Dur31 contributes to the capacity of *C. albicans* to actively alkalinize its environment under nutrient starvation by importing amine-containing substrates (e.g. spermidine) and thereby contributing to the intracellular production of ammonium which is then released as volatile ammonia by the Ato proteins (Figure 8). In line with its alkalinization function, expression of *DUR31* has been shown to be down-regulated at alkaline pH *in vitro* [63]. However, it is unlikely that this gene is constitutively repressed at alkaline pH, as Dur31 was required for filamentous growth in RPMI medium, which is neutral/alkaline. Indeed, we originally identified *DUR31* as being transcriptionally upregulated during oral candidiasis and Dur31 was required for oral epithelial damage, suggesting that this transporter also functions at neutral/alkaline pH.

In summary, we have characterized the roles of 12 previously unknown function genes in oral infection. For one gene (*DUR31*) we provide evidence for multiple cellular and pathogenic functions including histatin 5 import, polyamine utilization, environmental alkalinization, hyphal morphogenesis, epithelial and endothelial destruction, immune evasion and virulence (Figure 8).

## Materials and Methods

### Ethics statement

All animal experiments were in compliance with the German animal protection law and were approved (permit no. 03-007/07) by the responsible Federal State authority (Thüringer Landesamt für Lebensmittelsicherheit und Verbraucherschutz) and ethics committee (beratende Komission nach § 15 Abs. 1 Tierschutzgesetz). The use of human primary cells in this study was conducted according to the principles expressed in the Declaration of Helsinki. All protocols used in this study were approved by the local ethics committee of the University of Jena under the permit no. 2207-01/08. Written informed consent was provided by all study participants.

### Strains and culture conditions

All *C. albicans* strains used in this study are listed in Table 2. The triple-auxotrophic strain BWP17 complemented with plasmid CIp30 [9] was used as wild type control in all experiments. Strains were routinely cultivated on YPD agar [1% yeast extract, 2% bacto-peptone, 2% D-glucose, 2% agar] or SD minimal medium agar [2% dextrose, 0.17% yeast nitrogen base, 0.5% ammonium sulfate, 2% agar]. Liquid overnight cultures were grown in YPD or SD medium in a shaking incubator at 30°C and 180 rpm. For selection purposes fungal cells were spread on SD agar supplemented with 20 µg ml^−1^ arginine, histidine and/or uridine as required. *E. coli* was grown on LB agar [1% bacto-tryptone, 0.5% yeast extract, 1% NaCl, 2% agar]. Overnight liquid cultures of *E. coli* were cultivated in a shaking incubator at 37°C and 210 rpm. For growth curves of *C. albicans* strains, overnight YPD cultures grown at 30°C were diluted to an OD_600_ of 0.1 in a 200 µl final volume of the desired medium. Growth of the strains was then recorded by measurement of the OD_600_ at 30 min interval for up to 50 hours in an ELISA reader (Infinite M200, Tecan) [64]. Experiments were performed at least twice in triplicate.

### Strain construction

The *dur31*Δ/Δ homozygous null mutant was constructed using a PCR-based gene disruption technique [65]. Using the Arg-, His- and Ura-auxotrophic strain BWP17 [66] as background, the complete open reading frames (ORFs) of both *DUR31* alleles were replaced with polymerase chain reaction (PCR)-amplified *ARG4* and *HIS1* disruption cassettes flanked by 104 base pairs of target homology region. Two sequential transformations using the improved lithium-acetate method [67] were applied for both disruption cassettes. Primers DUR31-FG and DUR31-RG (Table S2) were used for generation of the deletion cassettes with the pFA-*ARG4* and pFA-*HIS1* plasmids as templates. Resultant deletion cassettes were used to sequentially delete both copies of *DUR31* (orf19.6656). The resultant Ura-auxotrophic mutant was rendered prototrophic for uridine by transformation with the NcoI-linearized plasmid CIp10, which harbours the *URA3* gene and stably integrates at the *RPS10* locus [68]. The correct deletion of both alleles and integration of CIp10 was verified by colony PCR using target gene and disruption/integration cassette flanking and internal primers: DUR31-F1, DUR31-R1, ARG4-F1, ARG4-R1, HIS1-F1, HIS1-R1, URA3-F2 and RPF-F1 (Table S2), respectively. An identical strategy with relevant gene-specific primers was employed for deletion and confirmation of orf19.1150, orf19.1353, orf19.2959.1, orf19.3617, orf19.3872, orf19.5443, orf19.5848, orf19.6200, orf19.6847, orf19.7670 and orf19.988.

Additionally, Southern blot analysis (Figure S5) using a 354 base-pair PCR product, generated with the primers DUR31-F2 and DUR31-R2 (Table S2) from *C. albicans* SC5314 genomic DNA, as a probe on HindII-digested genomic DNA was used to confirm deletion of *DUR31*/orf19.6656.

For construction of a *dur31*Δ/Δ::*DUR31*-reconstituted strain, the open reading frame of *DUR31* as well as 504 base pairs of upstream and 460 base pairs of downstream sequence were amplified from SC5314 genomic DNA with the Phusion High-Fidelity DNA Polymerase Kit (Finnzymes) using the *Hind*III restriction site containing primers DUR31rec-F1 and DUR31rec-R1 (Table S2). The PCR product was first digested with *Hind*III and then further purified with the QIAquick PCR Purification Kit (Qiagen). In parallel 0.3 µg µl^−1^ of plasmid CIp10 was digested with *Hind*III and the restriction enzyme then heat inactivated by incubation at 65°C for 20 min. The linearized plasmid was dephosphorylated with calf intestinal alkaline phosphatase (New England BioLabs) and gel extracted using the QIAquick Gel Extraction Kit (Qiagen). The *DUR31* insert and CIp10 vector were then ligated for 30 min at 22°C using the Rapid DNA Ligation Kit (Fermentas). Five µl of ligation product was used for the transformation of *E. coli* DH5α and positive clones were selected on LB agar plates supplemented with 50 µg ml^−1^ Ampicillin. Plasmid CIp10 carrying *DUR31* was re-isolated using plasmid miniprep (peqlab) and midiprep (Qiagen) kits and confirmed by control digestions with *Hind*III, *Sac*I and *Spe*I. The final plasmid was then digested with *Nco*I prior to transformation into the uridine auxotrophic *C. albicans* strain *dur31*Δ/Δ*ura^−^* (Table 2). Positive clones were selected on SD agar plates without amino acids. Correct integration was verified by PCR on whole yeast colonies using primers RPF-F1 and URA3-F2 (Table S2).

### Stress susceptibility

Aliquots of YPD overnight cultures were washed twice in phosphate buffered saline (PBS) and 10-fold serial dilutions in 5 µl (covering a range of 10^6^–10^1^ cells) were spotted onto SD agar containing 0.4 mM menadione (Sigma), 2 mM H_2_O_2_ (AppliChem), 1.5 M NaCl (Roth), 0.75 mM silver nitrate (Roth) or 450 µg ml^−1^ Congo red (Sigma) and incubated at 37°C for 3–4 days. Plates incubated at 42°C were photographed after 4–6 days. UV-stress was induced by exposure of 10-fold serial dilutions on YPD agar to 0, 2, 4 or 8 mJ/cm^2^ UV-C light with a wavelength of 254 nm using a UV-crosslinker (Bio-Link, Vilber-Lourmat). Plates were then incubated for 2 days at 37°C. Each experiment was repeated at least twice. Representative pictures are shown.

### Histatin 5 fungicidal assay

Sensitivities of *C. albicans* strains to histatin 5 were investigated by microdilution assay as previously described [59], [69].

### FITC-labeling of histatin 5

Labeling of histatin 5 (Sigma) was performed as previously described [70], [71]. Briefly, 1.25 µl of freshly dissolved FITC (1 mg/ml) in DMSO was added to 500 µl of histatin 5 (65 µM) in 50 mM potassium phosphate buffer. Following 16 h incubation at 4°C in darkness, residual FITC was inactivated by incubation with 10 µl 1 M NH_4_Cl for 2 h at 4°C in darkness. Fifty µl aliquots of FITC-labeled histatin 5 (FITC-histatin 5) were stored at −20°C until use.

### Histatin 5 localization study

To investigate intracellular localization of FITC-histatin 5, strains were grown overnight in YPD medium, washed twice with 10 mM sodium phosphate buffer (NaPB) and cell numbers were adjusted to 10^6^ cells ml^−1^ in 10 mM NaPB. Cells were incubated with 30 µM FITC-histatin 5 for 15 min at 30°C with shaking (300 rpm). Cells were then immediately mounted on glass coverslips and analyzed by fluorescence microscopy. Experiments were performed twice in duplicate. For quantification, the mean fluorescent intensities of at least 80 cells per strain were determined.

### Filamentation

Filamentation was investigated on solid water agar supplemented with 10% fetal bovine serum or 5% RPMI, on SLAD agar, on solid Spider medium [72] or by embedding in YPS agar [1% yeast extract, 2% bacto-peptone, 2% D(+)-saccharose, 2% agar]. RPMI agar and SLAD agar plates were incubated for 4 days, serum agar plates for 2 days, and Spider agar plates for 10 days at 37°C. Embedded plates were incubated at 25°C for 5 days. Experiments were performed twice in duplicate yielding similar results. Representative pictures are shown.

For analysis of filament formation on a single cell level, fungal cells were grown overnight to stationary phase in SD medium, washed twice with water, and resuspended in water. Cell numbers were adjusted to 10^4^ cells per well in a 24-well cell culture plate in RPMI1640 or water supplemented with 10% serum, and incubated at 37°C for 4 hours in the presence of 5% CO_2_. Experiments were performed in duplicate and repeated twice. Representative pictures are shown.

Induction of filamentation using host cells was performed by preparation of a monolayer of oral epthelial cells (TR146) as described previously [12] and infecting it with 10^5^ *C. albicans* cells. Monolayers were incubated at 37°C for three hours in a 5% CO_2_ atmosphere and hyphal cells were then differentially stained according to the invasion assay protocol described below. Representative pictures are shown.

For induction of hyphal microcolonies, fungal cells were grown overnight to stationary phase in YPD medium, washed twice with PBS, and resuspended in PBS. Cell numbers were adjusted to 10^4^ cells per ml and 10 µl (100 cells) were inoculated in 500 µl RPMI medium without serum per well in a 24-well cell culture plate and incubated at 37°C for 24 hours in presence of 5% CO_2_ in an incubator (Binder). Experiments were performed in quadruplicate on two different occasions. The dimensions of 40 cells per strain and experiment were determined using an inverse microscope (Leica DMIL) and the software LAS (Leica Application Suite). Representative pictures taken at 40× magnification are shown.

### Extracellular alkalinization assays

Investigation of extracelluar alkalinization by *C. albicans* was performed as previously described [17], with minor modifications. Briefly, for alkalinization on solid media, strains were grown on GM-BCG (1% yeast extract, 30 mM CaCl_2_, 3% glycerol, 0.01% bromocresol green, 4% agar) without glucose. Alkalinization assays in liquid media were conducted using medium 199 with Earle's salts (PAA) and supplemented with sodium hydrogen carbonate (PAA), according to the manufacturer's instructions. All media were adjusted to pH 4 using HCl and NaOH. For assays on solid GM-BCG media, 12- or 6-well plates were used, in which 2 or 5 ml of molten GM-BCG agar, without glucose, were added and allowed to solidify. *C. albicans* strains were grown to stationary phase overnight in YPD, centrifuged, washed once in water and diluted to an OD_600_ of 1.0 in water. Seven µl of this dilution were then pipetted onto the agar, with one strain per well. Plates were incubated at 30°C and alkalinization was followed daily for up to 14 days as a progressive change of the medium colour from green to blue. Experiments were performed at least in duplicate and repeated three times. Representative pictures are shown. Alkalinization assays in liquid medium 199 (M199) were performed in 96-well plates. Overnight YPD fungal cultures were harvested by centrifugation, washed once in water and adjusted to an OD_600_ of 1.0 in pH 4-adjusted medium 199. Serial five-fold dilutions were prepared and plates were incubated at 37°C for 24 h. Alkalinization of the medium was observed by a change of color from yellow to red. Experiments were performed in duplicate on three different occasions. Representative pictures are shown. In order to directly quantify the alkalinization capacity of the different strains, overnight YPD cultures were adjusted to OD 0.1 in M199 pH 4 and 8 ml were added per well to a 6-well cell culture plate and incubated at 37°C for 24 hours. The cells were then resuspended and the pH and OD_600_ was measured.

### Western blot analysis

Western blot analysis for detection of phosphorylated Mkc1 was performed as previously described [73], with some modifications. Briefly, overnight YPD cultures of the BWP17+CIp30 wild type and the *dur31*Δ/Δ, *dur31*Δ/Δ::*DUR31* and *mkc1*Δ/Δ mutant strains were adjusted to OD 0.5 in 10 ml final volume and grown under the following conditions for 4 hours at 30°C with shaking (180 rpm): (i) SD minimal medium, and (ii) SD minimal medium with 450 µg/ml Congo red. Cells were collected by centrifugation at 4°C and washed twice with cold lysis buffer containing 1× PBS, 3 mM KCl, 2.5 mM MgCl_2_, 0.1% Triton X-100, 50 mM NaF, 2 mM Na_3_VO_4_. Cell pellets were resuspended in cold lysis buffer (see above) containing a protease inhibitor cocktail (Roche). Cells were then mechanically disrupted by adding acid-washed glass beads and bead beating in a Precellys 24 homogenizer (peqlab). Protein concentrations were determined by BCA Protein Assay (Pierce). Protein samples (80 µg) were mixed with one-fourth volume of 4× sample buffer containing 125 mM Tris-HCl (pH 6.8), 50% glycerol, 4% SDS, 2.5% β-mercaptoethanol, and 0.02% bromophenol blue for SDS-PAGE. Samples were heated at 95°C for 5 min and then separated by SDS-PAGE using 12% acrylamide gels. Proteins were electro-transferred to Protran B85 nitrocellulose membranes (Whatman) and blocked with 5% BSA (Serva) in PBS with 0.05% tween. Blots were then probed with primary anti-phospho-p44/42 MAP kinase antibody (1∶1000, Cell Signaling Technology) and secondary goat anti-rabbit-horseradish peroxidase (HRP)-conjugated antibody (1∶2500, Santa Cruz), and developed using the Enhanced Chemiluminescent (ECL) SuperSignal West Dura kit (Thermo Scientific) according to the manufacturers' instructions. Membranes were then stripped for 30 min at 50°C using a buffer containing 2% SDS, 125 mM Tris-HCl (pH 6.8) and 0.7% β-mercaptoethanol. Stripped membranes were then blocked with 5% BSA (Serva) in PBS with 0.05% tween and re-probed for α-tubulin (loading control) by using a primary rat anti-α-tubulin antibody (1∶1000, AbD Serotec) and a secondary goat anti-rat HRP-conjugated antibody (1∶2000, Santa Cruz), and developed as described above. Experiments were performed twice.

Western blot analysis for detection of Ssa1/2 levels was performed as described above, with minor modifications. The phosphatase inhibitors NaF and Na_3_VO_4_ were omitted from the lysis buffer (see above). Blots were probed with primary mouse anti-Hsp70 monoclonal antibody (1∶1000, Stressgen) and secondary goat anti-mouse HRP-conjugated antibody (1∶2500, Santa Cruz).

### Surface expression of Ssa1/2

The assessment of surface expression of Ssa1/2 was performed based on a previously described method [15], with some modifications. Briefly, to analyze the surface distribution of Ssa1/2 on the different strains, 10^5^ yeast cells in 1 ml RPMI 1640 medium with L-glutamine and HEPES were added to 12 mm diameter glass cover slips in a 24-well cell culture plate. Following a 90 min incubation in 5% CO_2_ at 37°C, the resulting germ tubes were washed three times with PBS and fixed with 4% paraformaldehyde for 1 hour at room temperature (RT), washed again twice with PBS, and blocked with 2% BSA for 30 min at RT. Following blocking, the germ tubes were then washed again three times in PBS, and then stained with a mouse anti-Hsp70 monoclonal antibody (1∶100, Stressgen) and Alexa Fluor 555-conjugated goat anti-mouse IgG (1∶500, Invitrogen) as the secondary antibody. Next, the germ tubes were counter stained with Alexa Fluor 647-conjugated concanavalin A (1∶500, Invitrogen) to label the cell surface. The glass coverslips were mounted inverted on microscope slides and imaged by fluorescence microscopy. Experiments were performed twice in duplicate.

### Endothelial and oral epithelial cells

The human buccal carcinoma epithelial cell line TR-146 (Cancer Research Technology, London) [74] and the human umbilical vein endothelial cell line HUVEC (ATCC CRL-1730, LGC Standards, Promocell) were cultured and passaged in Dulbecco Modified Eagles Medium (DMEM) with 2 mM L-glutamine (PAA) supplemented with 10% heat inactivated (56°C, 10 min) fetal bovine serum (FBS, PAA). For experiments, TR146 cells were used during passage 10–20 and HUVEC cells during passage 10–40. Both cell lines were cultured in an incubator at 37°C with 5% CO_2_ atmosphere. Cultivation medium was replaced by fresh medium every second day and accutase (PAA) was used for detaching cells after confluency had reached about 80–100%.

### Quantification of adherence to host cells

*C. albicans* adherence studies were performed using ibidi μ-Slides VI ^0.4^ with six channels per slide. For adherence assays with human host cells, 1.8×10^4^ endothelial or epithelial cells were seeded per μ-slide channel and incubated for 3 days at 37°C and 5% CO_2_ with medium changed daily. Confluent monolayers were infected with 1.5×10^4^ *C. albicans* cells per channel for 45 min. Monolayers were then thoroughly washed with PBS to remove un-adhered fungal cells and fixed with 4% paraformaldehyde. *C. albicans* cells were subsequently stained with calcofluor white for 30 min in the dark and visualized by fluorescence microscopy (Leica DM5500B, Leica DFC360 FX). The number of adhered cells was determined by counting at least 50 high power fields of 200 µm×200 µm size. Experiments were performed in duplicate on two separate occasions.

### Quantification of invasion into host cells

The invasion capacity of the different *C. albicans* strains was determined as previously described [30]. Briefly, epithelial TR146 cells were grown to confluency on 15 mm diameter glass coverslips for 2–3 days. Monolayers were washed with PBS and infected with 10^5^ *C. albicans* yeast cells for 3 hours at 37°C and 5% CO_2_. Next, epithelial cells were washed twice with PBS and fixed with 4% paraformaldehyde (Roth). Fungal cells were then stained for 45 min with fluorescein-conjugated concanavalin A (Con A) (Invitrogen). After washing with PBS, epithelial cells were permeabilized in 1% Triton X-100 for 15 min. Next, fungal cells were stained with calcofluor white for 30 min. All incubation steps were carried out in the dark. Coverslips were then rinsed three times with water and mounted with the cells upside down on microscope slides with ProLong Gold Antifade Reagent. Fluorescence microscopy was performed (Leica DM5500B, Leica DFC360 FX) using appropriate filter sets for detection of fluorescein-conjugated Con A and calcofluor white. At least 100 *C. albicans* cells were examined for each strain and the invasion rate was expressed as percentage of invaded cells divided by the number of invaded plus non-invaded cells. Representative pictures were taken for each strain. All experiments were performed in duplicate on two separate occasions.

### Quantification of damage to host cells

The extent of damage caused to host cells by the *C. albicans* strains was quantified by measuring lactate dehydrogenase (LDH) activity with the Cytotoxicity Detection Kit (Roche Applied Science). TR146 or HUVEC cells were adjusted to 10^5^ cells ml^−1^ in DMEM with 10% FBS and 200 µl were seeded per well in 96 well plates (TPP). Plates were incubated at 37°C and 5% CO_2_ for 2 days until confluency had been reached. Cells were then washed twice with PBS and 100 µl DMEM with 2% FBS were added per well. For the *C. albicans* strains, aliquots of overnight YPD cultures were washed twice in PBS, diluted to 5×10^5^ cells ml^−1^ in DMEM without FBS and 100 µl used for infection of host cells. Controls included a medium only control, a low control with uninfected host cells and a high control with uninfected host cells and medium supplemented with 1% Triton X-100 prior to measurement. Incubation was carried out at 37°C and 5% CO_2_ for 15 or 24 h. Measurement of LDH activity with the Cytotoxicity Detection Kit was performed according to the manufacturer's manual. Absorbance of the samples was measured at 490 nm. Medium only and low control values were subtracted from all sample values. Damage was expressed as percentage of the high control, which was set to 100%. Each experiment was performed at least twice in triplicate.

### Susceptibility to killing by neutrophils

Neutrophils were isolated from blood of healthy human donors by a density gradient centrifugation using Histopaque 1077 and 1119 (Sigma, MO, USA) following the manufacturer's instructions. Polymorphonuclear cells (PMNs) were obtained after a centrifugation step at 700 g for 30 min at room temperature and then transferred to PBS. The remaining erythrocytes were lysed in a lysis buffer (0,83% NH_4_Cl, 10 mM HEPES, pH 7.0), the PMNs washed once in PBS and resuspended in 1 ml RPMI1640+5% FBS. For investigating susceptibility of *C. albicans* to killing by neutrophils, 100 µl of fungal overnight cultures were collected and washed twice with PBS. *C. albicans* cells were opsonized with 50% human serum for 30 min at 37°C. Following centrifugation and resuspension in PBS, 10^5^ cells ml^−1^ were inoculated into RPMI1640+5% FBS. Neutrophils and fungal cells were then mixed in a ratio of 10∶1 (final volume: 400 µl) and incubated for 3 hours at 37°C in the presence of 5% CO_2_. Neutrophils were lysed by the addition of 100 µl 0.25% SDS at 30°C in order to release phagocytosed *C. albicans* cells. After addition of 900 µl cold water and 20 U of DNase-1, cells were incubated for 15 min at 30°C. Following preparation of appropriate dilutions, aliquots were spread in duplicate on YPD and incubated for 24 hours at 37°C. Three independent experiments were performed.

### Murine model of hematogenously disseminated candidiasis

Five to six weeks old female Balb/C mice (*Mus musculus*) (18–20 g; Charles River, Germany) were used for the experiments. The animals were housed in groups of five in individually ventilated cages and cared for in strict accordance with the principles outlined in the *European Convention for the Protection of Vertebrate Animals Used for Experimental and Other Scientific Purposes* (http://conventions.coe.int/Treaty/en/Treaties/Html/123.htm). Mice were challenged intravenously on day 0 with 5×10^5^ cfu in 200 µl PBS via the lateral tail vein. The health status of the mice was examined at least twice a day by a veterinarian. Body surface temperature and body weight were recorded once a day. Mice showing severe signs of illness like isolation from the group, apathy, hypothermia and drastic weight loss, were anaesthetized by application of 200 µl ketamine hydrochloride (50 mg ml^−1^) prior to blood collection by heart puncture. Gross pathological alterations were recorded during necropsy. For histology, left kidneys were collected and fixed with buffered formalin and paraffin-embedded sections were stained with Periodic acid-Schiff (PAS) according to standard protocols.

### Statistical analysis

Differences in damage of endothelial and oral epithelial cells were compared by two-tailed Student's t-test. The statistical analysis for the susceptibility of *C. albicans* strains to killing by neutrophils was performed using Turkey's Multiple Comparison test. Differences in survival of mice were evaluated by Log-rank (Mantel-Cox) and Gehan-Breslow-Wilcoxon tests. *P*-values≤0.05 were considered to be statistically significant. All statistical tests were performed using GraphPad Prism version 5.00.

### Accession numbers for genes and proteins mentioned in the text (NCBI Entrez Gene ID number)

*C. albicans*: orf19.1150 (3645322); orf19.1353 (3648169); orf19.2959.1 (no data); *GTR1* (3643914); orf19.3872 (3644865); *BNA4* (3640528); orf19.5848 (3647594); orf19.6200 (3639533); *DUR31* (3646965); orf19.6847 (3646149); orf19.7670 (3638948); orf19.988 (3647183); *DUR3* (3644760); *SSA1* (3636229); *SSA2* (3644711); *MKC1* (3639710); *ATO5* (3643652).

## Supporting Information

Figure S1 ***dur31*** **Δ/Δ has normal adherence and invasion properties upon contact with oral epithelial cells.** (A) Adherence assays were performed using ibidi μ-Slides VI^0.4^. Confluent epithelial cell monolayers were infected with 1.5×10^4^ *C. albicans* cells for 45 min. Monolayers were then thoroughly washed 3× with PBS to remove non-adhered fungal cells and fixed with 4% paraformaldehyde. *C. albicans* cells were subsequently stained with calcofluor white and quantified by fluorescence microscopy. The number of adhered cells was determined by counting at least 50 high power fields of 200 µm×200 µm size. Results are the mean ± SEM of two independent experiments, each performed in duplicate. (B) Invasion of *dur31*Δ/Δ mutant cells into human-derived oral epithelial cells is comparable to that of the wild type. Monolayers of confluent epithelial cells were infected with 10^5^ *C. albicans* yeast cells and incubated for 3 hours at 37°C and 5% CO_2_. After washing with PBS, cells were fixed with 4% paraformaldehyde. Fungal cells were then stained for 45 min with fluorescein-conjugated concanavalin A (ConA). Epithelial cells were then permeabilized with 1% Triton X-100. Next, fungal cells were stained with calcofluor white. Fluorescence microscopy was performed using appropriate filter sets for detection of Con A (stains only the extracellular, non-invaded fungal elements) and calcofluor white (stains both invaded and non-invaded fungal elements). At least 100 *C. albicans* cells were examined for each strain and the invasion rate was expressed as percentage of invaded cells divided by the number of invaded plus non-invaded cells. Results are the mean ± SEM of two independent experiments, each performed in duplicate.
(TIF)

Figure S2 ***dur31*** **Δ/Δ exhibits normal filament formation in liquid media and upon contact with oral epithelial cells.** (A) Wild type (Wt), *dur31*Δ/Δ and *dur31*Δ/Δ::*DUR31* filament formation in liquid RPMI and 10% serum. Overnight cultures were diluted 1∶2500 into RPMI1640, and water supplemented with 10% fetal bovine serum in 24-well cell culture plates and incubated at 37°C in presence of 5% CO_2_. Cells were photographed after 4 h. Scale bar: 20 µm. (B) Filament formation on epithelial monolayers. TR146 epithelial cells were cultured to confluency and infected with *C. albicans* wild type (Wt) and *dur31*Δ/Δ cells for three hours. Fungal cells were then differentially stained with fluorescein-conjugated ConA and calcofluor white, and visualized by fluorescence microscopy. Representative pictures are shown. Scale bar: 25 µm.
(TIF)

Figure S3 **Expression levels of Ssa1/2 on the surface of** ***dur31*** **Δ/Δ are comparable to those of the wild type.** Wild type (Wt) and *dur31*Δ/Δ mutant cells were grown in RPMI medium at 37°C and 5% CO_2_ for 90 min, washed, fixed and then stained with a mouse anti-Hsp70 monoclonal antibody (primary) and an Alexa Fluor 555-conjugated goat anti-mouse antibody (secondary). Cells were counterstained with Alexa Fluor 647-conjugated concanavalin A to label the cell surface. (A) Fluorescent microscopic images of anti-Hsp70 antibody and anti-*C. albicans* cell surface antibody. The merged images show co-localization (yellow) of the anti-Hsp70 antibody and anti-*C. albicans* cell surface antibody. (B) Fluorescent intensity of different cross sections of the filaments shown in the merged images in panel (A). The green lines represent the fluorescent intensity of the anti-Hsp70 antibody and the red lines represent the fluorescent intensity of the anti-*C. albicans* cell surface antibody. The letters (a–d) denote the positions of the cross sections in panel (A) at which the fluorescent intensities were measured. (C) Western blotting with an anti-Hsp70 antibody for detection of Ssa1/2 (two bands) in cell wall extracts of the wild type (Wt) and *dur31*Δ/Δ mutant. The blot was stripped and re-probed for α-tubulin as a loading control.
(TIF)

Figure S4 **Phylogenetic relatedness of** ***C. albicans*** **orf19.6656 (Dur31) with other orthologous proteins and the urea transporter Dur3.** The tree was generated using the Clustal W method in the DNASTAR Lasergene MegAlign sequence analysis software. All sequences were retrieved from CGD's (*Candida* Genome Database) Multi-Genome Search database and SGD's (*Saccharomyces* Genome Database) Fungal Genomes Search database using *C. albicans* orf19.6656 (Dur31) or orf19.781 (Dur3) as protein query sequence. Selected fungal species encoding Dur3 are shown. *C. dubliniensis* CD36_53230; *C. albicans* orf19.6656; *C. tropicalis* CTRG_05438; *C. parapsilosis* CPAG_05452; *L. elongisporus* LELG_03888; *D. hansenii* DEHA2E22396g; *P. stipitis* PICST_60304; *C. lusitaniae* CLUG_04732; *C. glabrata* CAGL0I08613g; *K. lactis* KLLA0C11913g; *C. immitis* CIMG_00418; *N. crassa* NCU01977.1; *M. globosa* MGL_3550; *U. maydis* UM02953.1. Asterisks indicate putative *C. albicans* Dur3 orthologues. *A. nidulans* AN0418; *A. terreus* ATEG_02629; *A. niger* An01g03790; *A. fumigatus* ureA (Dur3); *A. clavatus* ACLA_029800; *M. oryzae* MGCH7_ch7g226; *S. pombe* SPBC23G7.13c; *C. gattii* CGB_J2070C. Dur31 orthologues of CUG-clade species are marked in red. Species marked in green were selected amongst the best hits to the *C. albicans* Dur3 (orf19.781) protein sequence. *C. albicans* Dur31 is marked in bold.
(TIF)

Figure S5 **Deletion of both** ***DUR31*** **(orf19.6656) alleles in** ***C. albicans*** **.** The correct deletion of *DUR31* was confirmed by Southern blot analysis. Strains BWP17 (Wt), *dur31*Δ, *dur31*Δ/Δ*ura^−^* and *dur31*Δ/Δ were analyzed. A 354 base-pair (bp) PCR product, with *C. albicans* SC5314 genomic DNA as template, was used as a probe on *Hind*II-digested genomic DNA. (A) Expected band sizes are: 2927 bp (wild type *DUR31*), 1692 bp (*ARG4*-deletion-cassette) and 2173 bp (*HIS1*-deletion-cassette). (B) Southern blot.
(TIF)

Table S1 **Transcriptional upregulation of** ***C. albicans*** **unknown function genes during oral** ***in vivo*** **and** ***in vitro*** **infections.**
(DOC)

Table S2 **Primers used in this study.**
(DOC)
