# Supplementary figures and images for: The Novel Candida albicans Transporter Dur31 Is a Multi-Stage Pathogenicity Factor
Source: PLoS Pathog. 2012 Mar 15;8(3):e1002592. doi: 10.1371/journal.ppat.1002592 (PMC3305457; doi:10.1371/journal.ppat.1002592)

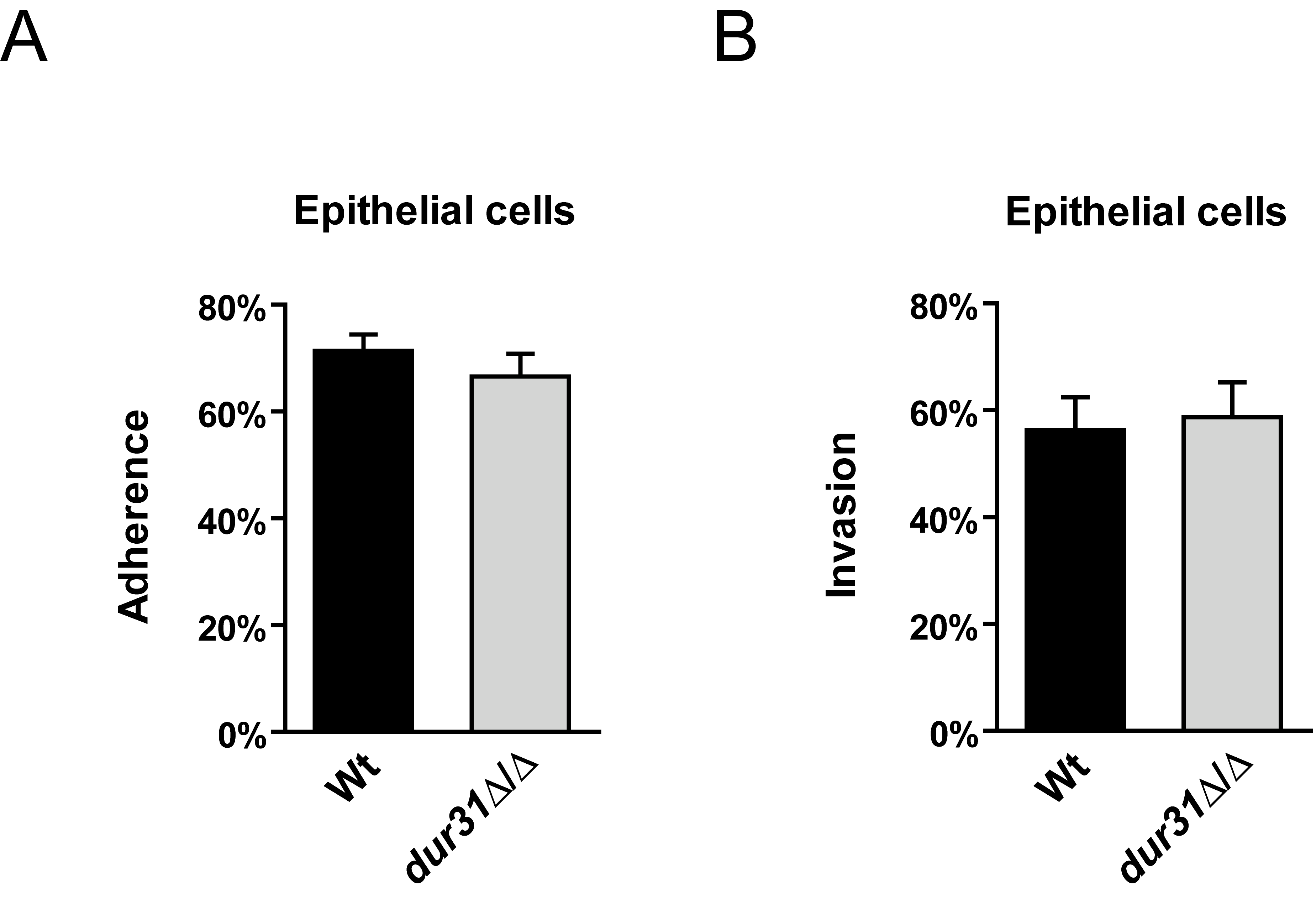

Supplement: Figure S1 — dur31 Δ/Δ has normal adherence and invasion properties upon contact with oral epithelial cells. (A) Adherence assays were performed using ibidi μ-Slides VI0.4. Confluent epithelial cell monolayers were infected with 1.5×10^4^ C. albicans cells for 45 min. Monolayers were then thoroughly washed 3× with PBS to remove non-adhered fungal cells and fixed with 4% paraformaldehyde. C. albicans cells were subsequently stained with calcofluor white and quantified by fluorescence microscopy. The number of adhered cells was determined by counting at least 50 high power fields of 200 µm×200 µm size. Results are the mean ± SEM of two independent experiments, each performed in duplicate. (B) Invasion of dur31Δ/Δ mutant cells into human-derived oral epithelial cells is comparable to that of the wild type. Monolayers of confluent epithelial cells were infected with 105 C. albicans yeast cells and incubated for 3 hours at 37°C and 5% CO2. After washing with PBS, cells were fixed with 4% paraformaldehyde. Fungal cells were then stained for 45 min with fluorescein-conjugated concanavalin A (ConA). Epithelial cells were then permeabilized with 1% Triton X-100. Next, fungal cells were stained with calcofluor white. Fluorescence microscopy was performed using appropriate filter sets for detection of Con A (stains only the extracellular, non-invaded fungal elements) and calcofluor white (stains both invaded and non-invaded fungal elements). At least 100 C. albicans cells were examined for each strain and the invasion rate was expressed as percentage of invaded cells divided by the number of invaded plus non-invaded cells. Results are the mean ± SEM of two independent experiments, each performed in duplicate. (TIF) [file ppat.1002592.s001.tif]

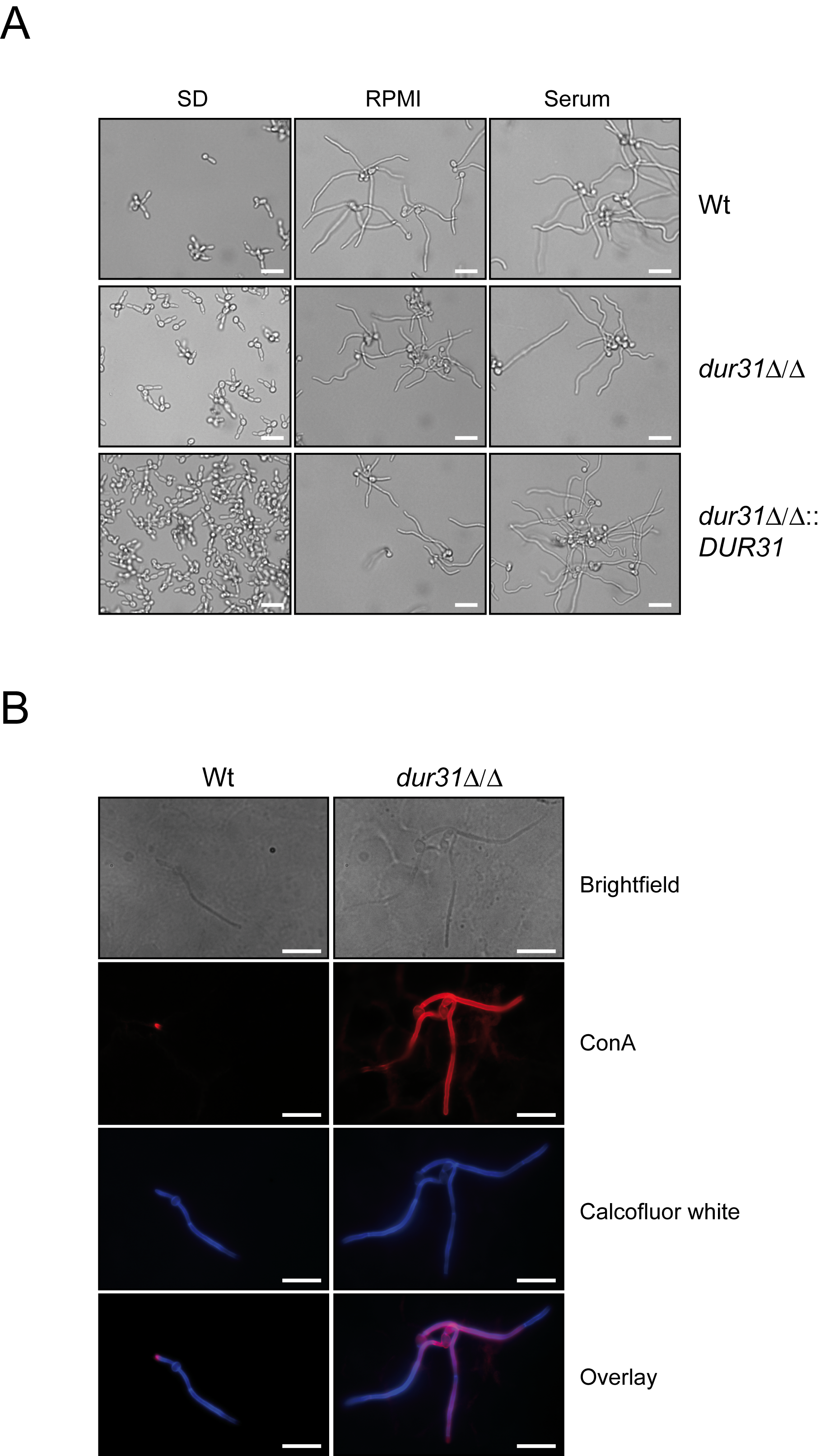

Supplement: Figure S2 — dur31 Δ/Δ exhibits normal filament formation in liquid media and upon contact with oral epithelial cells. (A) Wild type (Wt), dur31Δ/Δ and dur31Δ/Δ::DUR31 filament formation in liquid RPMI and 10% serum. Overnight cultures were diluted 1∶2500 into RPMI1640, and water supplemented with 10% fetal bovine serum in 24-well cell culture plates and incubated at 37°C in presence of 5% CO2. Cells were photographed after 4 h. Scale bar: 20 µm. (B) Filament formation on epithelial monolayers. TR146 epithelial cells were cultured to confluency and infected with C. albicans wild type (Wt) and dur31Δ/Δ cells for three hours. Fungal cells were then differentially stained with fluorescein-conjugated ConA and calcofluor white, and visualized by fluorescence microscopy. Representative pictures are shown. Scale bar: 25 µm. (TIF) [file ppat.1002592.s002.tif]

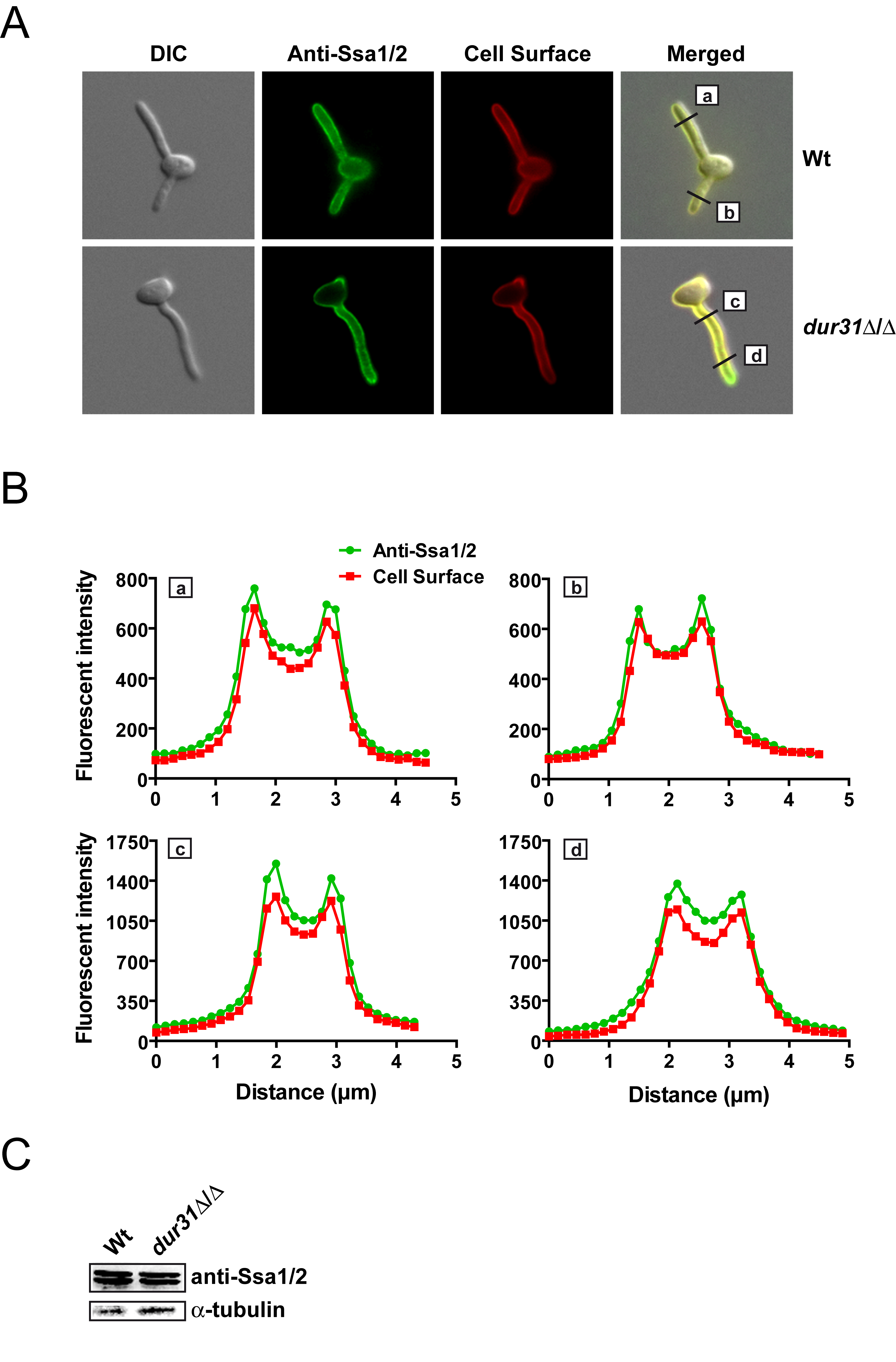

Supplement: Figure S3 — Expression levels of Ssa1/2 on the surface of dur31 Δ/Δ are comparable to those of the wild type. Wild type (Wt) and dur31Δ/Δ mutant cells were grown in RPMI medium at 37°C and 5% CO2 for 90 min, washed, fixed and then stained with a mouse anti-Hsp70 monoclonal antibody (primary) and an Alexa Fluor 555-conjugated goat anti-mouse antibody (secondary). Cells were counterstained with Alexa Fluor 647-conjugated concanavalin A to label the cell surface. (A) Fluorescent microscopic images of anti-Hsp70 antibody and anti-C. albicans cell surface antibody. The merged images show co-localization (yellow) of the anti-Hsp70 antibody and anti-C. albicans cell surface antibody. (B) Fluorescent intensity of different cross sections of the filaments shown in the merged images in panel (A). The green lines represent the fluorescent intensity of the anti-Hsp70 antibody and the red lines represent the fluorescent intensity of the anti-C. albicans cell surface antibody. The letters (a–d) denote the positions of the cross sections in panel (A) at which the fluorescent intensities were measured. (C) Western blotting with an anti-Hsp70 antibody for detection of Ssa1/2 (two bands) in cell wall extracts of the wild type (Wt) and dur31Δ/Δ mutant. The blot was stripped and re-probed for α-tubulin as a loading control. (TIF) [file ppat.1002592.s003.tif]

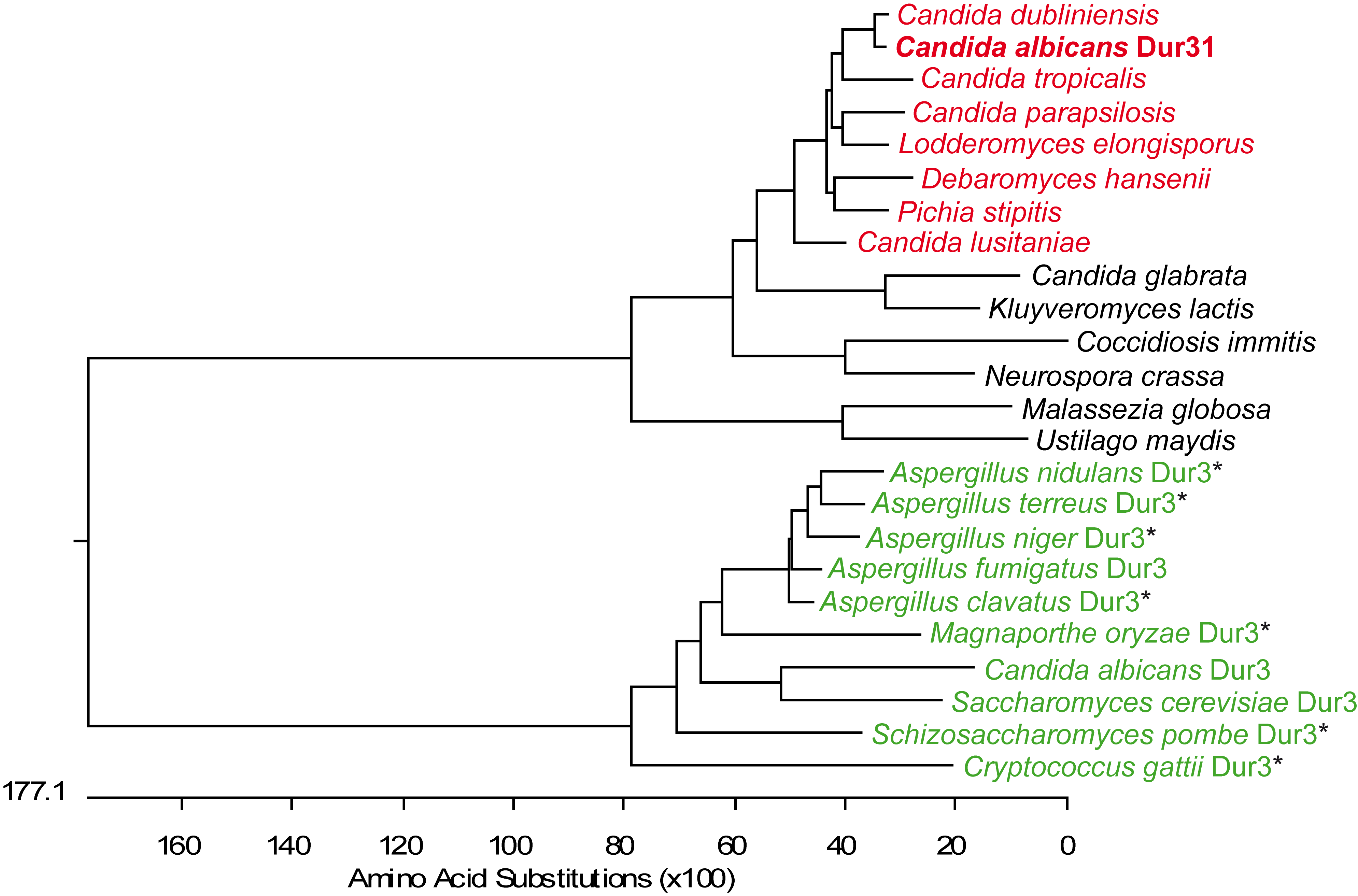

Supplement: Figure S4 — Phylogenetic relatedness of C. albicans orf19.6656 (Dur31) with other orthologous proteins and the urea transporter Dur3. The tree was generated using the Clustal W method in the DNASTAR Lasergene MegAlign sequence analysis software. All sequences were retrieved from CGD's (Candida Genome Database) Multi-Genome Search database and SGD's (Saccharomyces Genome Database) Fungal Genomes Search database using C. albicans orf19.6656 (Dur31) or orf19.781 (Dur3) as protein query sequence. Selected fungal species encoding Dur3 are shown. C. dubliniensis CD36_53230; C. albicans orf19.6656; C. tropicalis CTRG_05438; C. parapsilosis CPAG_05452; L. elongisporus LELG_03888; D. hansenii DEHA2E22396g; P. stipitis PICST_60304; C. lusitaniae CLUG_04732; C. glabrata CAGL0I08613g; K. lactis KLLA0C11913g; C. immitis CIMG_00418; N. crassa NCU01977.1; M. globosa MGL_3550; U. maydis UM02953.1. Asterisks indicate putative C. albicans Dur3 orthologues. A. nidulans AN0418; A. terreus ATEG_02629; A. niger An01g03790; A. fumigatus ureA (Dur3); A. clavatus ACLA_029800; M. oryzae MGCH7_ch7g226; S. pombe SPBC23G7.13c; C. gattii CGB_J2070C. Dur31 orthologues of CUG-clade species are marked in red. Species marked in green were selected amongst the best hits to the C. albicans Dur3 (orf19.781) protein sequence. C. albicans Dur31 is marked in bold. (TIF) [file ppat.1002592.s004.tif]

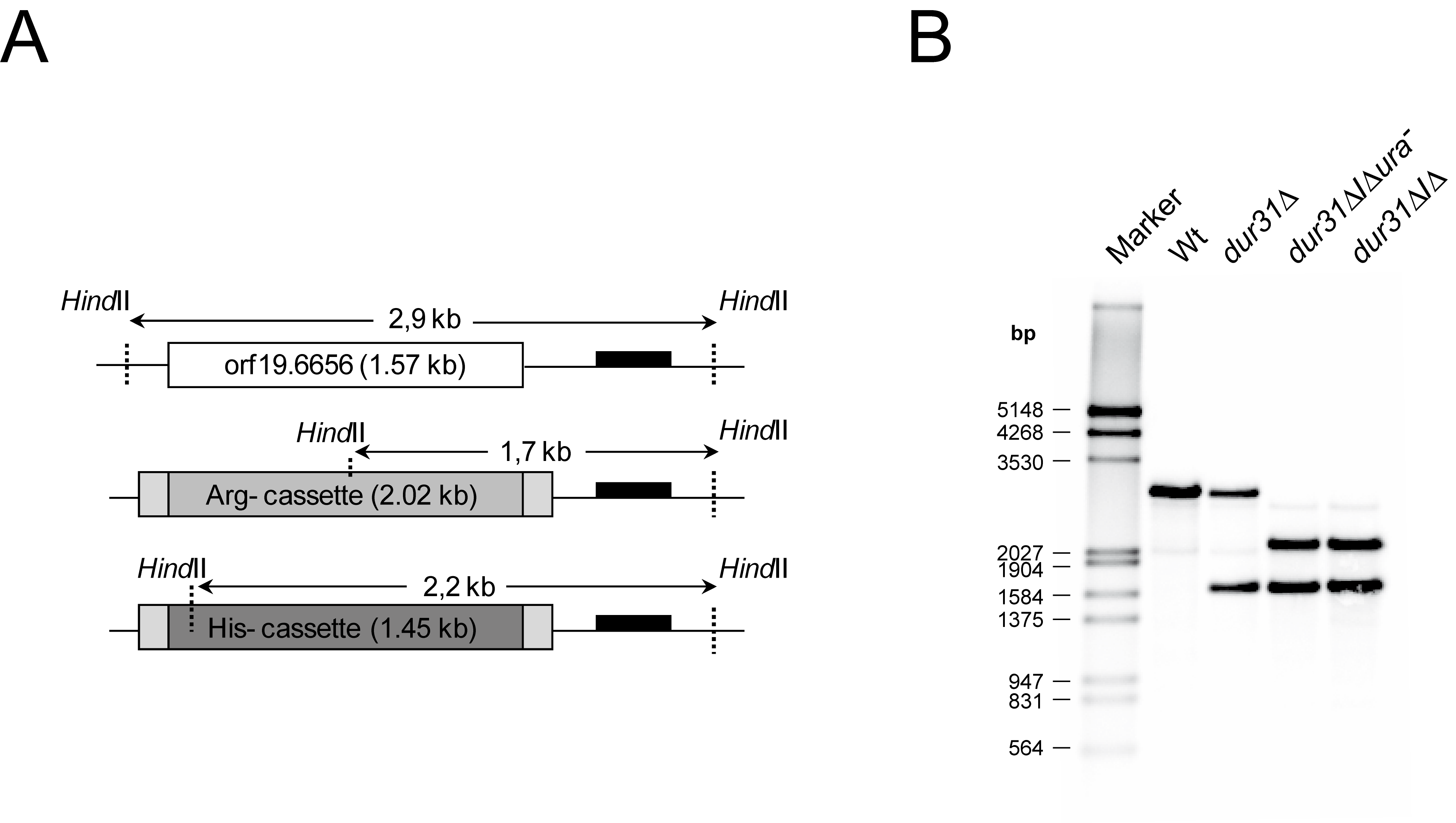

Supplement: Figure S5 — Deletion of both DUR31 (orf19.6656) alleles in C. albicans . The correct deletion of DUR31 was confirmed by Southern blot analysis. Strains BWP17 (Wt), dur31Δ, dur31Δ/Δura− and dur31Δ/Δ were analyzed. A 354 base-pair (bp) PCR product, with C. albicans SC5314 genomic DNA as template, was used as a probe on HindII-digested genomic DNA. (A) Expected band sizes are: 2927 bp (wild type DUR31), 1692 bp (ARG4-deletion-cassette) and 2173 bp (HIS1-deletion-cassette). (B) Southern blot. (TIF) [file ppat.1002592.s005.tif]
