# Supplementary material for: The Novel Candida albicans Transporter Dur31 Is a Multi-Stage Pathogenicity Factor
Source: PLoS Pathog. 2012 Mar 15;8(3):e1002592. doi: 10.1371/journal.ppat.1002592 (PMC3305457; doi:10.1371/journal.ppat.1002592)
Supplement: Table S1 — Transcriptional upregulation of C. albicans unknown function genes during oral in vivo and in vitro infections. (DOC) [file ppat.1002592.s006.doc]

**Table S1. Transcriptional upregulation of *C. albicans* unknown function genes during oral *in vivo* and *in vitro* infections.**

|  |  | **RHE** | | | | |  |
| --- | --- | --- | --- | --- | --- | --- | --- |
| **Gene** | **Patient** | **1 h** | **3 h** | **6 h** | **12 h** | **24 h** | **Reference** |
| orf19.1150 | 1.03 | 0.88 | 2.27 | 2.05 | 0.30 | 1.29 | [1] |
| orf19.1353 | n.d. | 2.63 | 1.22 | 0.05 | 11.24 | 3.65 | [1] |
| orf19.2959.1 | 1.29 | 0.45 | 1.01 | 0.74 | 0.20 | 2.26 | [1] |
| orf19.3617 | 2.36 | 1.59 | 2.89 | 2.17 | 1.30 | 1.40 | [1] |
| orf19.3872 | n.d. | n.d. | 1.45 | 2.99 | 9.07 | 0.12 | [1] |
| orf19.5443 | 1.10 | 1.94 | 1.94 | 4.32 | 3.47 | 1.36 | [1] |
| orf19.5848 | 1.27 | 2.50 | 1.36 | 2.54 | 2.51 | 2.76 | [1] |
| orf19.6200 | n.d. | 1.06 | 1.74 | 0.64 | 5.50 | 2.27 | [1] |
| orf19.6656 | 2.00 | 0.93 | 1.02 | 1.12 | 0.94 | 1.09 | [1] |
| orf19.6847 | 1.33 | 1.96 | 1.52 | 3.54 | 2.77 | 1.53 | [1] |
| orf19.7670 | 1.32 | 0.92 | 0.71 | 1.89 | 3.87 | 1.96 | [1] |
| orf19.988 | 3.01 | 2.02 | 1.09 | 0.92 | 3.49 | 1.00 | [1] |

A transcriptional gene induction ≥ 2.00 was considered a significant upregulation.

1. Zakikhany K, Naglik JR, Schmidt-Westhausen A, Holland G, Schaller M, et al. (2007) In vivo transcript profiling of Candida albicans identifies a gene essential for interepithelial dissemination. Cell Microbiol 9: 2938-2954.
