# Supplementary material for: The Novel Candida albicans Transporter Dur31 Is a Multi-Stage Pathogenicity Factor
Source: PLoS Pathog. 2012 Mar 15;8(3):e1002592. doi: 10.1371/journal.ppat.1002592 (PMC3305457; doi:10.1371/journal.ppat.1002592)
Supplement: Table S2 — Primers used in this study. (DOC) [file ppat.1002592.s007.doc]

**Table S2.** Primers used in this study.

| **Primer** | **Sequence (5’→ 3’)** | **Reference** |
| --- | --- | --- |
| DUR31-FG | aatcaaaaagaaagaaagagcaaactttaaattatcatcattttcattcttcatcccttgattttgttcaagtcatataatcccaatcacaagattctatataagaagcttcgtacgctgcaggtc | This study |
| DUR31-RG | tgcaggaaagagaaatttaaataggaagaataatacttcagacttaatgccatatgattaatatacaaaaaatatttacgttgtcatacccttctgacgtcttatctgatatcatcgatgaattcgag | This study |
| DUR31-F1 | gcctggttgtttgttgaatttcgc | This study |
| DUR31-R1 | caccgccaaagcctaacaccaac | This study |
| DUR31-F2 | gttggtgttaggctttggcggtg | This study |
| DUR31-R2 | ggacctccagataatagacaccagaag | This study |
| DUR31rec-F1 | ggtatgtggtgttaagcttagcaag | This study |
| DUR31rec-R1 | gatggtaagcttatgagttgagaag | This study |
| orf19.1150-FG | agctgatcacatacaaaatctttttctccatccccacattcacataaccatacacttgcacatacacatacacatctattcctattatcatagttcataccctcgaagcttcgtacgctgcaggtc | This study |
| orf19.1150-RG | tgaaatggatatgaatggaaaataaataaatagacaggaaaagatagcaacatacaagaaacatcatcacgtaagaattagaaaaacaaaggctcatttgatcatctgatatcatcgatgaattcgag | This study |
| orf19.1150-F1 | ccttcctttagtttctactccatc | This study |
| orf19.1150-R1 | tcgaaagttgatactgtggaagc | This study |
| orf19.1353-FG | gaaaaagaaaccaaataaacaacaacaattgaaaaatcatcaatgaatgatgatttgtatgggtttcaaaggggggtgtgtggaaaaagcaatttgtttatgagaagcttcgtacgctgcaggtc | This study |
| orf19.1353-RG | tccattggtttaatattgtctaaatctaatggtaaagcttcagacttagactttttctttcccgccatgatcgttaatttgtgtatttatttaaaaataggaatctgatatcatcgatgaattcgag | This study |
| orf19.1353-F1 | aaaatttcagtgattataattac | This study |
| orf19.1353-R1 | tgatgatgatcttgttttag | This study |
| orf19.2959.1-FG | caattcaattccccctaagtagtctctttttttttttgattctctcttttctattttataatcacttctttttttatttcttacaagtacattttactatcaaagaagcttcgtacgctgcaggtc | This study |
| orf19.2959.1-RG | attcatttaaataataaagaaaagcaattttcctttttcatcgtcacaattgttaaccgtactgctgctaaattgtgcaatacatttaccttgttatatgaccgtctgatatcatcgatgaattcgag | This study |
| orf19.2959.1-F1 | gttaaagagtcacgcacttggttac | This study |
| orf19.2959.1-R1 | ctacaacacaataccagcatagtgac | This study |
| orf19.3617-FG | atttctatttaaatttaaattattgaaaacaaaacaatctcacttttttcatcttataactaaaataaattaaatattaccattatacacacacattcttcttatcgaagcttcgtacgctgcaggtc | This study |
| orf19.3617-RG | tacaaaaagattaaaagttgattggttggttggctggttctttgggttggtatgtacttgtatacattcctcttctacttgatcctcaattcttgcttgctgtttctgatatcatcgatgaattcgag | This study |
| orf19.3617-F1 | gtagcgctaccaatataagtcgtg | This study |
| orf19.3617-R1 | ctcccacctgttaagtttgactc | This study |
| orf19.3872-FG | atcaatcacaagttggctcaactcttttcttttcttttattttgtttatttaccaattatatcaacttcacttatagctagccctctctgattgctattgatcagaagcttcgtacgctgcaggtc | This study |
| orf19.3872-RG | cttagaatctacagtaatactgaaatggcaatgatgtaaatatataaccagtaacaacacaataatatactgtttttttctctctgtgcaccacttttctaaactctgatatcatcgatgaattcgag | This study |
| orf19.3872-F1 | ctgtatatagtaaattccacctgtaac | This study |
| orf19.3872-R1 | aatgtgtttggtatcgtcggaataatc | This study |
| orf19.5443-FG | aacaaagctgagaattgtgtatgtgtgtataaaaattcgagagagagaaaaaaaaaaatggttcgacttttattgtaaaacctttctttaaccacaccactgttgaagcttcgtacgctgcaggtc | This study |
| orf19.5443-RG | catggtttggtatactctaaaaacaaaatatgtattactatgtacaactagttggtcaaaaaaattttttcagtaccaaagaaaatttaaccaccccacgactttctgatatcatcgatgaattcgag | This study |
| orf19.5443-F1 | ctacagaactcaatagaagagaag | This study |
| orf19.5443-R1 | caggctgggcctttattatac | This study |
| orf19.5848-FG | tttatatagtcagattatctttttttttttaaatacagcacctccgcattatcaagaccacaacactcaaataatcacttcatttcttccattgccttattccagaagcttcgtacgctgcaggtc | This study |
| orf19.5848-RG | tactacccggggcagtaagtgagtaagtcggcgataaaactacacaacaacattgattcaccaatcataattaaatattatcaaaatattaaaaactatacacatctgatatcatcgatgaattcgag | This study |
| orf19.5848-F1 | caattgttgtcaattcgtccaaattc | This study |
| orf19.5848-R1 | tatatactaataacacaatcgccttg | This study |
| orf19.6200-FG | gtttttcatatacaagattttcttctctttataaacacaattgttgaattaacaaaaactattaagtggataaaagagataaaagcaaaagaagaactgtcgaagcttcgtacgctgcaggtc | This study |
| orf19.6200-RG | taaaaaaggaaacaaatcacaattcaatggaacaaaaacaacaagcagctctagaaacaatttcatctctccaccatccatctcctctccttcgtaaccaatctgatatcatcgatgaattcgag | This study |
| orf19.6200-F1 | catgatgccaccattagcttc | This study |
| orf19.6200-R1 | ccatccgtaaggaacagc | This study |
| orf19.6847-FG | ctcttgaaccacattaataaccaactctgaaaaactgtacaccaagctatcaactcagatacatataaacgtatctatatatcacattcgtttcactttccaatgaagcttcgtacgctgcaggtc | This study |
| orf19.6847-RG | cacacaagcagacatgaatgttattcaactactcacaaaggctaatctttctattatttatccagtctacatgctatatatgacttttaaaataaacaccactttctgatatcatcgatgaattcgag | This study |
| orf19.6847-F1 | cgcctatgttttcacgtgacttg | This study |
| orf19.6847-R1 | ctaactgtcctaacaaccattacc | This study |
| orf19.7670-FG | ttaggtagttaaatataaactttttttttttttctctatttaaggggtgaaactgtaaacaaaaaaaaaatttcttcgggttctttctcgtcacttaccaacccgaagcttcgtacgctgcaggtc | This study |
| orf19.7670-RG | attaagggtaattacaagttggtacttacaaatgtcgacaaagacagtaaagacgctttgtctccatacgaggctagaatgtatgtagttgattaaatatacatctgatatcatcgatgaattcgag | This study |
| orf19.7670-F1 | ggaagtggtgctacagcattcag | This study |
| orf19.7670-R1 | gtggtgaggatgtcaagtttgagc | This study |
| orf19.988-FG | cacatatctaccatatcaacatagagaagaaccccccctcctcacacaacatgtcttcattatcaagaagcaactcaactggttcagtaccataccttgacgaagcttcgtacgctgcaggtc | This study |
| orf19.988-RG | caaataaatagccaggtaaataaataagtgaatgaacaaatttgttggataaaaataaacacatttaaaagaatgttctcgatagaatgacgtttctcaactctgatatcatcgatgaattcgag | This study |
| orf19.988-F1 | acttttttcttttttacttttc | This study |
| orf19.988-R1 | acggattatgtaaagacaaac | This study |
| ARG4-F1 | ggatatgttggctactgatttagc | [1] |
| ARG4-R1 | aatggatcagtggcaccggtg | [1] |
| HIS1-F1 | ggacgaattgaagaaagctggtgcaaccg | [1] |
| HIS1-R1 | caacgaaatggcctcccctaccacag | [1] |
| URA3-F2 | ggagttggattagatgataaaggtgatgg | [1] |
| RPF-F1 | gagcagtgtacacacacacatcttg | This study |

Underlined nucleotides of forward generation (FG) and reverse generation (RG) primers indicate pFA-*ARG4* and pFA-*HIS1* annealing regions, and underlined nucleotides of primer DUR31rec-F1 and DUR31rec-R1 indicate HindIII restriction sites.

1. Martin R, Hellwig D, Schaub Y, Bauer J, Walther A, et al. (2007) Functional analysis of Candida albicans genes whose Saccharomyces cerevisiae homologues are involved in endocytosis. Yeast 24: 511-522.
